# Polyphenol Extract from Evening Primrose (*Oenothera paradoxa*) Inhibits Invasion Properties of Human Malignant Pleural Mesothelioma Cells

**DOI:** 10.3390/biom10111574

**Published:** 2020-11-19

**Authors:** Malgorzata Chmielewska-Kassassir, Katarzyna Sobierajska, Wojciech M. Ciszewski, Malgorzata Bukowiecka-Matusiak, Dorota Szczesna, Izabela Burzynska-Pedziwiatr, Wieslaw Wiczkowski, Waldemar Wagner, Lucyna A. Wozniak

**Affiliations:** 1 Department of Structural Biology, Medical University of Lodz, Zeligowskiego 7/9 Str., 90-752 Lodz, Poland; malgorzata.chmielewska-kassassir@umed.lodz.pl (M.C.-K.); malgorzata.bukowiecka-matusiak@umed.lodz.pl (M.B.-M.); dorota.szczesna@umed.lodz.pl (D.S.); izabela.burzynska-pedziwiatr@umed.lodz.pl (I.B.-P.); 2 Department of Molecular Cell Mechanisms, Medical University of Lodz, Mazowiecka 6/8 Str., 92-215 Lodz, Poland; katarzyna.sobierajska@umed.lodz.pl (K.S.); wojciech.ciszewski@umed.lodz.pl (W.M.C.); 3 Institute of Animal Reproduction and Food Research of Polish Academy of Sciences, Tuwima 10 Str., 10-748 Olsztyn, Poland; w.wiczkowski@pan.olsztyn.pl; 4 Laboratory of Cellular Immunology, Institute of Medical Biology PAS, 106 Lodowa St., 93-232 Lodz, Poland; wwagner@cbm.pan.pl

**Keywords:** malignant pleural mesothelioma, evening primrose extract, *Oenothera paradoxa* Hudziok extract, polyphenols, cytotoxicity, invasion

## Abstract

Extracts from the defatted evening primrose (*Oenothera paradoxa* Hudziok) seeds are the source of a range of stable polyphenolic compounds, including ellagic acid, gallic acid, and catechin. Our studies evaluate, for the first time, the influence of evening primrose isopropanol extract (EPE) on malignant pleural mesothelioma (MPM) cells. MPM is rarely diagnosed, its high aggressiveness and frequently noted chemoresistance limit its treatment schemes and it is characterized by low prognostic features. Here, we demonstrate that EPE inhibited MPM growth in a dose-dependent manner in cells with increased invasion properties. Moreover, EPE treatment resulted in cell cycle arrest in the G2/M phase and increased apoptosis in invasive MPM cell lines. Additionally, EPE strongly limited invasion and MMP-7 secretion in MPM cancer cells. Our original data provide evidence about the potential anti-invasive effects of EPE in MPM therapy treatment.

## 1. Introduction

Although phytotherapy has been used in human cultures from ancient ages, a renewed interest has been observed in the last two to three decades. Phytochemicals demonstrate a wide range of biological activities, such as anti-inflammatory abilities, neuroprotection, antioxidant effect, and even cancer prevention and treatment [1,2]. Of particular interest are polyphenols that are abundantly detected in numerous extracts from selected plants. So far, it has been demonstrated that many chemically different polyphenols, including resveratrol, epigallocatechin-3-gallate (EGCG), and derivatives of catechin (e.g., quercetin) can inhibit cancer cells proliferation, angiogenesis, and metastasis [3]. The extracts obtained from defatted seeds of *Oenothera* sp., including *O. paradoxa,* contain biologically active chemicals, such as gallic acid (GA), ellagic acid (EA), or penta-*O*-galloyl-β-d-glucose (PGG). These compounds reveal potential antitumor effects manifested by the inhibition of cancer development [4,5,6]. Gallic acid is a natural plant triphenol. Both GA and its derivatives show antitumor activity against a variety of human cancer cells, including lung, breast, prostate, and skin lineages [7,8,9,10]. The mechanisms of GA action remain unclear. However, several different pathways seem to be involved such as cell-cycle arrest [7,8], caspase-3 and mitochondria-dependent cells death [11], or the direct inhibition of matrix metalloproteinases (MMPs), resulting in a loss of migration and invasion capacity of tumor cells [9,10,12]. Ellagic acid is a dimeric derivative of gallic acid, which has been tested against several cancer cell lines, including human lung and colon cancer cell lines [13,14]. EA represents strong anti-proliferative and anti-apoptotic effects in certain malignancies [15,16]. Several studies also pointed out EA anti-metastatic activity, including prostate and bladder cancers [17,18]. Other compounds identified in the Oenothera paradoxa extracts like quercetin or epigallocatechin-3-gallate inhibit cancer cell growth via the downregulation of anti-apoptotic factors (e.g., Bcl-2) and the induction of pro-apoptotic Bax, thus forcing cancer cells’ death [19,20]. It was earlier demonstrated that EGCG (catechin derivative) inhibited premalignant and malignant endothelial cells by angiogenesis suppression (through vascular endothelial growth factor expression arrest) [21] but also directly reduced the expression of specific MMPs [22]. Previous studies also confirmed an increase in the sensitivity of melanoma and hepatocellular carcinoma cell lines to vincristine chemotherapy when the cells were pre-treated with ethanolic *Oenothera paradoxa* extract [23]. Vincristine (VCR) is a plant alkaloid widely used in the treatment of solid tumors. The ethanolic extract from *O. paradoxa* seeds, rich in procyanidins and PGG, potentiated the action of VCR. Therefore, supplementing standard anticancer agents with natural extracts rich in polyphenolic compounds, including evening primrose extract (EPE), seems to be one of the promising solutions managing tumor cells.

Malignant pleural mesothelioma (MPM) is a relatively rare cancer with non-specific symptoms, but its aggressive course makes it a tumor with a poor prognosis. The median survival is only 4–12 months from the time of diagnosis of the advanced stage [24], and the disease brings a significantly worse prognosis in men, with a 6.7% 5-year-survival rate, in comparison to women, with a survival rate of 17.0% for that time [25]. The MPM occurrence is related to prolonged occupational exposure to mineral fibers like asbestos or erionite, which leads to chronic inflammation in mesothelial cells. The latency period is quite spread and may last from 20 to even 60 years [26], which results in the development of tumor cells able to metastasize and invade surrounding tissues. Due to the delayed and very often ambiguous diagnosis, MPM also becomes a serious challenge from a therapeutic perspective because of the resistance to conventional treatment [27]. Therefore, searching for new therapeutic strategies seems to be crucial for patients with MPM.

The present study aims to demonstrate the anticancer properties of a polyphenol-rich extract obtained from *Oenothera paradoxa* Hudziok on MPM tumor cell growth, migration, and invasion ability. 

## 2. Materials and Methods 

### 2.1. Chemicals and Reagents

LC-MS grade reagents were purchased from Witko (J.T. Baker, Philipsburg, NJ, USA). Cell culture reagents were purchased from Gibco (Thermo Fisher Scientific, Warsaw, Poland). Chemicals used in extract preparations were obtained from Avantor Performance Materials Poland S.A. (formerly POCH S.A., Gliwice, Poland). All other chemicals, including analytic standards of selected phenolic compounds were purchased from Sigma-Aldrich (Munich, Germany) unless stated otherwise. 

### 2.2. Plant Material and Extract Preparation

Post-industrial defatted evening primrose *(Oenothera paradoxa)* seeds were obtained from Agrofarm S.A. pharmaceutical company (Tuszyn, Poland). The polyphenol extract was obtained using a Soxhlet apparatus. Defatted milled evening primrose seeds were extracted with 60% (*v*/*v*) aqueous solution of the isopropanol at 105 °C for 6, 12, 24, and 48 h. The extract obtained after 24 h of extraction was used for further chemical analysis and biological studies. The ratio of plant material to the isopropanol solution was 1:6 (*w*/*v*). The obtained extract was concentrated in a rotary evaporator (Heidolph Hei-VAP, Germany), lyophilized, and stored for further analyses. The lyophilized extracts were stored in tightly sealed vials using thermoplastic parafilm that ensures protection from moisture. Vials were additionally packed in a plastic bag and stored in a fridge in 4 °C.

For total Polyphenol Content determination and cell culture studies, the dried extract was dissolved in 20% ethanol/phosphate-buffered saline (PBS) solution (*v*/*v*). For liquid chromatography mass spectrometry analysis, the extract was dissolved in 20% methanol.

### 2.3. Characterization of Polyphenol Extract

The total polyphenol content (TPC) was calculated using the Folin–Ciocalteu method [2] and expressed as gallic acid equivalents (GAE) [28]. The EPE concentration range 0.01–1000 µg/mL was equivalent to 0.00208–208.24 µg GAE/mL, that means 0.01224–1224 µM GAE. GAE is a commonly used determinant in assessing TPC reported in previous studies [29,30]. To calculate TPC in *O. paradoxa* extract, the gallic acid standard curve was determined for the concentration range from 12.5 to 200 µg/mL of gallic acid. Briefly, 2.5 mL of 10 times diluted Folin–Ciocalteu reagent was added to 0.5 mL of evening primrose extract solution in the concentrations: 100 µg/mL and 50 µg/mL and incubated with 2 mL of 7.5% (*w*/*v*) sodium carbonate solution for 2 h at room temperature. The absorbance was measured at 765 nm on a LAMBDA 25 UV Spectrophotometer (Perkin Elmer, UK). All determination was performed from three independent experiments. In this study, the concentration of extract used for in vitro MPM cells treatment was presented in µg/mL of extract as well as µM GAE to enable further discussion of the results. 

### 2.4. Analysis of Phenolic Profile

The qualitative characteristic of the phenolic profile for the examined *O. paradoxa* extract was performed using analytical liquid chromatography (ekspert™ microLC 200 System, Eksigent Technologies part of AB SCIEX, Singapore) conjugated with a mass spectrometer (TripleTOF 4600, AB SCIEX, Singapore) equipped with an electrospray ionization source and time-of-flight mass analyzer according to [31] method. 

A sample of the filtrated and diluted extract was injected onto a HALO Phenyl-Hexyl column (2.7 µm particles, 0.5 × 50 mm, Eksigent part of AB SCIEX, Dublin, CA, USA), with a separation program using a gradient of binary phases A and B containing 0.1% formic acid, consisting of water and acetonitrile, respectively. The flow rate was: 1 mL per minute, the 4 min run gradient was: 5% of solvent B to 99.4% in 3 min, and 88% in 4 min. Mass detection was performed on the quadrupole time-of-flight MS system operating in both negative (ESI−) and positive (ESI+) electrospray ionization mode. The ion spray voltage was set to −/+4.5 kV, respectively. Source and chromatographic column temperatures were set at 300 °C and 40 °C, respectively. Mass accuracy was maintained to four decimal places using Calibrant Delivery System with APCI negative/positive calibration solution serving as a lock mass during the real-time analysis. For MS scanning, data acquisition was set from 50 to 1350 *m*/*z*, and for MS/MS fragmentation of target ions, the collision energy was −25 eV. Standard and blank samples, containing respectively each of the interested phenolic compounds with known concentrations and extract solvent (20% MeOH + 0.1% FA), were also injected during the LC-MS analysis.

The pre-identified, based on MS/MS fragmentation ions and retention time, phenolic compounds were quantitatively determined using standards according to retention times and mass spectra. The quantitative analysis of *O. paradoxa* extract was carried out using analytical liquid chromatography (microLC 200, Eksigent, Vaughan, ON, Canada) conjugated with a mass spectrometer (TripleTOF 5600+, AB SCIEX, Vaughan, ON, Canada) according to the above defined conditions for both extract and selected phenol analytic standards. Mass spectral data were acquired using Analyst^®^ TF 1.6 Software (AB SCIEX). Data analysis was performed using PeakView 2.0 (AB SCIEX), while the quantitative analysis was done using MultiQuant 3.0 software. The calibration curves for this study have an R2 in the range from 0.9977–0.9999. The parameters of analytes detection was LOD = 0.0013–0.1402 mg/g, while LOQ = 0.0042–0.4034 mg/g. Analyses were performed in triplicates.

### 2.5. Cell Culture and Treatment

Human malignant pleural mesothelioma cell lines, NCI-H28, MSTO-H211, and JU77, were kindly provided by Peter Szlosarek, PhD from the Queen Mary University of London, UK. Cells were maintained in RPMI1640 supplemented with 10% fetal bovine serum (FBS), penicillin, and streptomycin at 37 °C in a humidified atmosphere with 5% CO_2_. The cells for EPE treatment were grown to 80% confluence at the end of the experiment within the RPMI medium supplemented with 5% FBS. EPE stock solution was prepared freshly for every test in 20% EtOH (*v*/*v* in PBS) and was added to cells to get the final ethanol concentration of 0.1% (*v*/*v*). Control conditions were performed within 0.1% ethanol alone. 

### 2.6. Sulforhodamine B Assay

The sensitivity of the cell lines to EPE was determined using the sulforhodamine B (SRB) assay, as described previously [32]. Briefly, exponentially growing cells seeded into a 96-well plate were treated with the appropriate EPE concentration. After 72 h of drug exposure, the cells were fixed with Carnoy’s solution, washed twice, dried, and stained with SRB. The absorbance was measured at 570 nm on an Infinite F50 microplate reader (Tecan). The results were calculated as a percentage of controls, and the IC50 values were calculated with the GraphPad Prism v 8.4 software (GraphPad Inc. San Diego, CA, USA) using a four-parameter nonlinear logistic regression.

### 2.7. Wound-Healing Migration Assay

The confluent cells growing on 12-well tissue culture plates were wounded with a sterile 0.2-mL pipet, and the detached cells were removed by washing twice with PBS. After wounding, images were captured immediately (time 0), and then the progression of migration was snapped immediately after scratch, and 8 h with the EVOS FLoid Cell Imaging Station (ThermoFisher Scientific, Waltham, MA, USA). The wounded area was quantified in ImageJ software (Bethesda, MD, USA). A minimum of four randomly selected fields was analyzed. The results were shown as a percentage of recovery (% R) from three identically treated plates using the equation: % R = [1 − (Tt/T_0_)] × 100], (T0 is the wounded area at 0 h; Tt is the wounded area 8 h post-injury].

### 2.8. Transwell Invasion Assay

The invasion studies were performed using Matrigel invasion chambers (24-well cell culture inserts containing 8 µm membrane with a uniform layer of Matrigel [Corning]). The lower chamber contained medium with 20% FBS as a chemoattractant. The cells were suspended in serum-free medium and plated onto the upper chamber (50 µL cell density of 2 × 10^6^/mL) according to the manufacturer’s recommendations. Cells were allowed to migrate for 8 h into the bottom chamber in a humidified incubator at 37 °C in 5% CO_2_. After incubation, the migrated cells present on the lower surface of the insert were stained/visualized with 1% crystal violet water solution photographed (Leica DM2000LED) and counted using NIH ImageJ analysis software. The cells were counted from five fields.

### 2.9. Apoptosis Analysis

The effect of EPE on apoptosis induction was performed on exponentially growing cells treated with EPE at the desired concentration for 48 h using Dead Cell Apoptosis Kit with Annexin V Alexa Fluor™ 488 and Propidium Iodide according to the manufacturer instruction (Invitrogen, Eugene, OR, USA). Samples were analyzed on LSR Fortessa (Becton-Dickinson, BD Biosciences, San Jose, CA, USA), and apoptosis was determined using BD FACS Diva version 8.0 software (Becton-Dickinson, BD Biosciences, San Jose, CA, USA).

### 2.10. Cell-Cycle Analysis

The cell cycle analysis was performed as described previously [33]. Briefly, exponentially growing cells were treated with EPE at the desired concentration for 24 h. Then, cells were harvested by trypsinization, washed twice in ice-cold PBS, and fixed in 70% ethanol. After storing at least 24 h at 4 °C, cells were stained with 50 µg/mL propidium iodide and 100 µg/mL RNase A solution for 30 min at 37 °C. Then, samples were analyzed on an LSR Fortessa (Becton-Dickinson, BD Biosciences, San Jose, CA, USA), and cell cycle phases were determined using ModFit LT version 5.0 software (Verity Software House, Topsham, ME, USA).

### 2.11. Statistical Analysis

All experiments were performed at least in three independent replicates. The differences between groups were analyzed using one-way analysis of variance (ANOVA) followed by Tukey’s test with GraphPad Prism Software v5.02 software (GraphPad Inc. San Diego, CA, USA). The results are presented as means ± standard error unless otherwise state and a *p*-value less than 0.05 defined statistical significance.

## 3. Results

### 3.1. Qualitative and Quantitative Composition of EPE Extract

**Figure 1:**
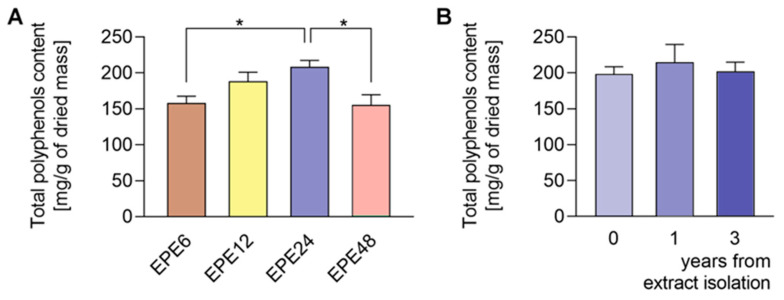
Determination of total polyphenols content (TPC) and its stability in evening primrose extracts (EPE) determined by the Folin–Ciocalteu method. (**A**) EPEs obtained in continuous Soxhlet extraction procedures performed for 6, 12, 24, and 48 h (EPE6, EPE12, EPE24, and EPE48, respectively). (**B**) Stability of EPE was determined by comparing the content immediately after extraction, as well as 1 and 3 years after extraction. TPC expressed in mg of GA per gram of dried extract. Data are means of three independent experiments ± SD. * *p* < 0.05 compared to control.

We estimated the amount of TPC in a gram of a dried extract and expressed their concentration in mg of gallic acid equivalents (GAE). The colorimetric Folin–Ciocalteu method of TPC quantification did show that EPE extracts isolated at different extraction times are characterized by different polyphenol content (Figure 1A). The *O. paradoxa* extract obtained in a continuous (24 h; EPE24) isopropanolic Soxhlet extraction procedure contains the highest number of polyphenols that are around 21% in a gram of dried extract mass (Figure 1A, Table 1A). The yield of extraction of EPE24 was 8.6% *w*/*w*). We observed that the shorter Soxhlet extraction times (6 and 12 h) do not allow for full saturation of EPE seeds with 60% isopropanol solvent during extraction cycles. In turn, the longer extraction time (48 h) at high temperature seems to be responsible for the degradation of polyphenolic compounds. Therefore, EPE24 with the highest TPC was selected for further research studies. Besides, TPC analysis in the dried mass showed that the EPE24 extract obtained by Soxhlet extraction maintained a constant level of polyphenols for at least three years (Figure 1B).

The EPE prepared in isopropanol (60% aqueous solution) by Soxhlet apparatus contained a broad range of polyphenol composition identified by LC-MS. The mass spectra example of MS scan (50–1350 *m*/*z*) obtained for EPE24 in negative ionization mode is shown in Figure S1 in Supplementary Information. Based on the parent molecular ion and fragmentation ions obtained in the MS/MS scan, we confirmed the presence of the in Table 1B indicated compounds. The qualitative LC-TripleTOF-MS analysis revealed polyphenols compounds belonging to different groups: flavan-3-ol (e.g., catechin, procyanidin dimers and trimers), hydrolysable tannins (e.g., penta-*O*-galloyl-β-d-glucose), as well as phenolic acids (e.g., protocatechuic and gallic acids) and flavonols (e.g., quercetin glucuronide) (Table 1B). 

Employing quantitative microLC-TripleTOF 5600^+^ mass spectrometer analysis, we confirmed the presence and determined the amount of low molecular weight compounds like ellagic acid, catechin, and gallic acid, present in highest quantity, but also their structural counterparts (epicatechin gallate, penta-*O*-galloyl-β-d-glucose and procyanidin B2) in a lower concentration, as indicated in Table 2. The chromatograms of the selected compounds are present in Figure S3 in Supplementary Information. Based on standard concentration curves and the peak area, the content of each confirmed phenolic compound was calculated per gram of the dried mass of isopropanolic *O. paradoxa* extract. Ellagic acid, gallic acid, and catechin constituted the majority of the discussed extract and referred to 37.3%, 24.7%, and 11.2% of total polyphenols present in EPE, respectively. Besides, *O. paradoxa* extract contained smaller but similar amounts of penta-*O*-galloyl-β-d-glucose (polyphenolic gallotannin) and epicatechin gallate (flavan-3-ol), 1.8% and 1.9% of extract TPC, respectively. In the present work, EPE was tested in in vitro studies within the concentration range from 100 to 300 µg/mL (that corresponds to the range from 20.8 to 62.5 µg GAE/mL or 122.3 to 364.4 µM GAE).

### 3.2. The Growth Inhibitory Effect of EPE on Mesothelioma Cells

**Figure 2:**
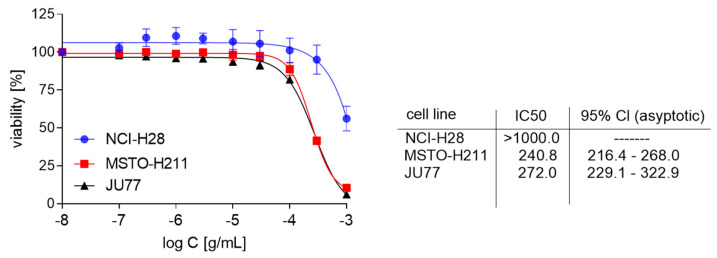
Determination of the growth inhibition by EPE. The data are presented as a percentage of the controls, the IC50 values were calculated using a four-parameter nonlinear logistic regression. Confidence intervals are shown as asymmetrical, due to the log transformation of the data required to perform IC50 calculations. Data are the means of three independent experiments ± SD, IC50 values are shown in µg/mL.

Here, we report our studies on the EPE effect on the proliferation of mesothelioma cells (MPM). Thus, three mesothelioma cell lines: NCI-H28, MSTO-H211, and JU77 were treated with different EPE concentrations for 72 h, and the SRB assay analyzed growth inhibitory effect. Our studies revealed that incubation with EPE decreased the proliferation of MPM cells in a dose-dependent manner. That effect was strongly marked in the MSTO-H211 and JU77 cell lines, with IC50 being 240.8 and 272 µg/mL, respectively (Figure 2). The NCI-H28 was only slightly affected by EPE treatment (IC50 > 1000 µg/mL).

### 3.3. EPE Induced Cell Cycle Arrest and Apoptosis in JU77 Cells

**Figure 3:**
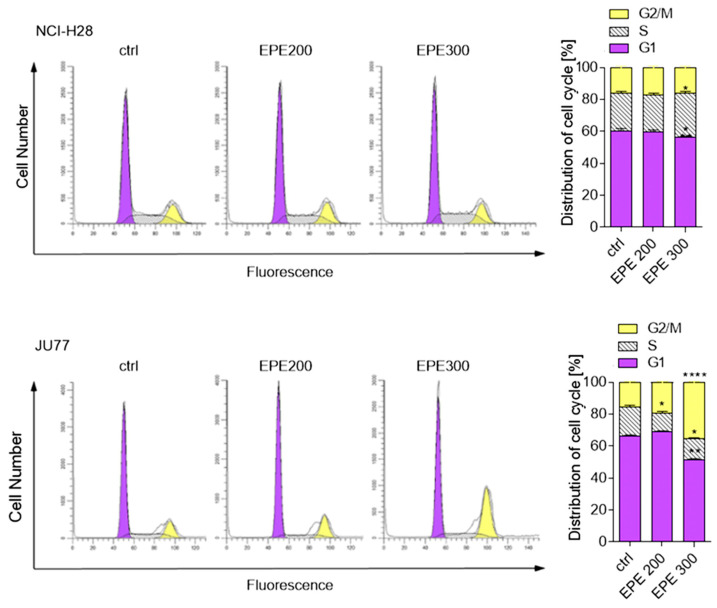
Effect of EPE on cell cycle distribution in malignant pleural mesothelioma (MPM) cells. Cells were treated with increasing concentration of EPE (200 µg/mL EPE200; 300 µg/mL EP300) for 24 h. Then, samples were submitted to flow cytometry analysis after propidium iodide (PI) staining. Histograms are representative of three independent experiments, and the graphs display the percentage of cells in the G1, S, and G2/M phases. Data are means of three independent experiments ± SD, * *p* < 0.05, ** *p* < 0.01, **** *p* < 0.001 compared to control.

Next, we examined whether the EPE-dependent growth inhibition would have consequences for the cell cycle distribution. We treated JU77 (EPE-sensitive) and NCI-H28 cells (EPE-low-sensitive) with an increasing EPE concentration for 24 h and analyzed the cell cycle phases FACS. As seen in Figure 3, EPE caused an increase in the G2/M only in JU77 cells. The highest used EPE concentration (300 µg/mL) increased 2.2 times the number of cells in the G2/M phase with a concomitant decrease in G1 and S phases compared to non-treated cells. In contrast, we did not observe any EPE effect on G2/M phase and only a slight increase in the S-phase in NCI-H28 cells (Figure 3).

**Figure 4:**
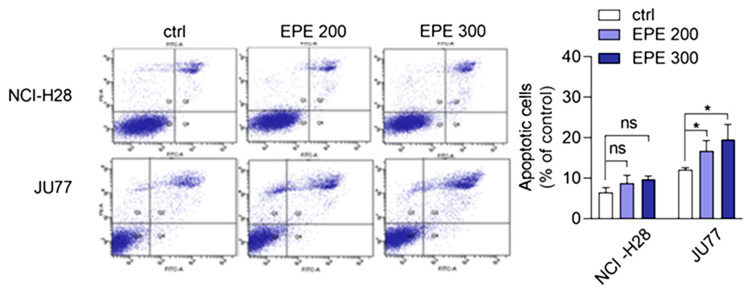
EPE-induced apoptosis in JU77 cells. NCI-H28 and JU77 cells were treated with increasing concentration of EPE (200 µg/mL EPE200; 300 µg/mL EPE300). After 48 h, cells were labelled with Annexin V-Alexa Fluor 488 and PI. The results are the mean value of the total apoptotic cells (the sum of early and late apoptotic cells) from three independent experiments ± SD, * *p* < 0.05 compared to control.

It is well known that the prolonged G2/M phase arrest might trigger a signaling pathway that induces cell death. Thus, we investigated whether EPE treatment induced apoptosis. The cells, treated as above for 48 h with EPE, were stained with Annexin V/PI solution and late apoptotic cells were quantified by FACS. We observed the concentration-dependent induction of apoptosis by EPE in JU77 cells (Figure 4). The highest EPE concentration (300 µg/mL) resulted in a 2.14-fold increase of apoptotic cells. In contrast, a slight increase of apoptotic cells at the highest EPE concentration for NCI-H28 cells was statistically insignificant. 

### 3.4. EPE Weakly Inhibited Cohort Cell Migration of MPM Cells Lines

**Figure 5:**
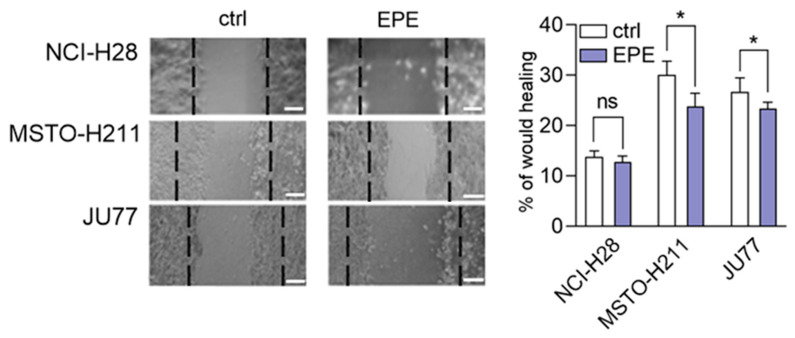
EPE-induced inhibition of wound healing in MSTO-H211 and JU77 cell lines. Analysis of wound healing was made immediately after scratch and 8 h later. The wounded areas were determined on the captured imaged by EVOS FLoid Cell Imaging Station in Image J software. Data are the means of three independent experiments ± SD, ns: non-statistical, * *p* < 0.05 compared to control.

The previous data suggested that similar to the oil from evening primrose; the seeds extract might regulate cell migration [34]. Therefore, the cohort cell migration ability of mesothelioma cells was measured by the wound healing assay. Cells were treated with a non-toxic dose of EPE (100 µg/mL) to avoid side effects of too high concentration of the extract. These experiments revealed that EPE treatment of the EPE highly-sensitive MPM cell lines (MSTO-H211 and JU77) caused an insignificant inhibition of cell migration after 8 h from wound formation (Figure 5). The MSTO-H211 cells showed less than 25% inhibition of cell migration, whereas the JU77 cells migrated about 10% slower in EPE presence. In contrast, the EPE non-sensitive NCI-H28 cell line did not show any effects of EPE treatment on cell migration (Figure 5).

### 3.5. EPE Treatment Caused a Lower Invasion of More Invasive MPM Cells Lines

**Figure 6:**
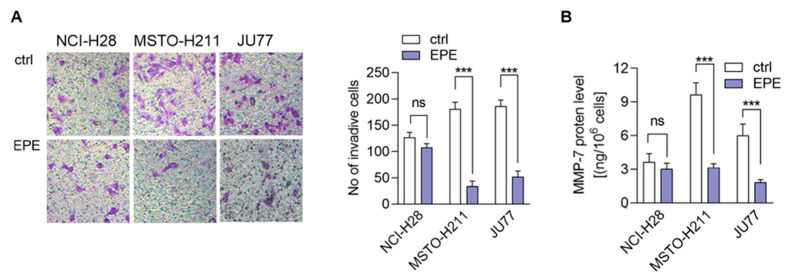
EPE inhibited the invasive ability of MSTO-H211 and JU77 cells. (**A**) The MPM pre-treated cells were put on the Boyden chamber with Matrigel^®^ placed on a 24-well plate. The cells migrated to the medium with 20% FBS that filled the lower part of wells for 8 hrs. Then it was fixed, labelled with 1% crystal violet, and captured. The analysis was made in ImageJ software. (**B**) The level of MMP-7 was determined in conditioned medium obtained from EPE-treated MPM cells. Data are the means of three independent experiments ± SD, ns: non-statistical, *** *p* < 0.005 compared to control.

It has been proposed previously that the extract from evening primrose might regulate the metalloproteinases (MMP) secretion in prostate and breast cancers [18,19,20,21,22]. Because MMPs are involved in the modulation of cancer invasion ability, we decided to examine whether EPE affects MPM cell invasiveness. As visible in Figure 6A, the MSTO-H211 and JU77 cells treatment with EPE (100 µg/mL) resulted in a 5-fold decrease of the invaded cell numbers. In contrast, the NCl-H28 cells were not sensitive to EPE treatment (Figure 6A). As we mentioned above, cancer cell invasion ability is regulated by the secretion of metalloproteinases (MMP). Therefore, we decided to compare the level of MMP-7 in the conditioned medium obtained from the MPM cells treated with EPE. On the basis of the ELISA assay, we detected a substantial decrease of the MMP-7 secretion by the MSTO-H211 and JU77 as a result of EPE treatment (Figure 6B). In particular, in the medium from MSTO-H211 cells, we detected a 3.07-fold decrease of the MMP-7 level and a JU77-3.27-fold decrease. In the NCI-H28 cells, an alteration of the MMP-7 level in the conditioned medium was not detectable (Figure 6B).

## 4. Discussion

In recent years, plant extracts are extensively studied to understand their ability to prevent the expansion of many chronic diseases, including cancer [35]. Among them, polyphenolic compounds play a pivotal role by regulating metabolic, inflammatory processes, and apoptosis [36]. Previous studies showed that the evening primrose (*Oenothera* sp.) extracts contain polyphenolic compounds, which present beneficial health effects [37,38,39]. The *Oenothera* sp. plants, among which *Oenothera biennis* and *Oenothera paradoxa* Hudziok are the most popular, are widely cultivated in Europe and Asia for edible oil pressing purposes and are rich with γ-linolenic acid [40]. Defatted seeds, obtained in large amounts as a by-product of this process, could serve as a potential material for further reprocessing. The amount of the waste material, inapt for animal fodder or any other utilization, allows for the extraction of valuable polyphenol compounds, which are the subject of numerous studies, due to their high biological activity. Recent findings showed the potential anticancer properties of polyphenol-rich extracts obtained from *Oenothera* sp. in several cancer types [29,30,41]. However, none has been established for the MPM tumor. Therefore, we aimed to develop the possibility of its supplementation that could support the existed therapies. For the present study, we obtained the polyphenol-rich extract (EPE) from *O. paradoxa* defatted seeds using the Soxhlet extraction, using the 60% aqueous isopropanol as a solvent. Then, we defined a group of phenolic compounds in EPE using LC-TOF-MS. Among many different chemical extraction procedures, including classical maceration, which relies on subsequent material overflowing with a solvent and pooling the obtained fractions, the Soxhlet extraction is a continuous process coupled with the circulation of boiling solvent, which ensures repeated contact of the extracted material with a new portion of solvent during each extraction cycle [42,43]. Its advantages are a short time, less solvent consumption, a larger sample mass to be extracted, a simple methodology, and convenience [44]. We performed the Soxhlet extraction of *O. paradoxa* seeds for different time points to obtain the highest TPC content, as longer extraction might lead to thermal decomposition of some thermolabile polyphenols [45]. Therefore, we recognized the optimum times of Soxhlet extraction of *O. paradoxa* seeds. Our studies revealed that the highest polyphenol concentration was observed after 24 h of the continuous extraction procedure. The elongation of extraction did not bring benefits in the form of increasing the polyphenol concentration, but on the contrary, resulted in a decrease of extracted polyphenol level. Herein, we confirmed in our 60% isopropanolic EPE extract all of the previously reported phenolic compounds [Table 1B]. In a previous study [29,30], the total polyphenol content determined by Folin–Ciocalteu method was 578.15 to 745.5 mg GAE/g higher than that observed in our extract (208.24 mg GAE/g). This discrepancy can be due to the different extraction methods of solid *O. paradoxa* as well as a different solvent, as we used isopropanol in contrast to ethanol, which has been used in previous studies [29,30]. However, in our opinion, both Soxhlet extraction and isopropanol used as a solvent impact on the unique properties of our extract, i.e., the potential antitumor effects against malignant pleural mesothelioma cells. The chemical composition of EPE reflects the presence of compounds belonging to different polyphenols classes including catechin and procyanidins (the latter being catechin derivatives belonging to flavan-3-ol group), penta-*O*-galloyl-β-d-glucose as well as di- and tetragalloyl glucose belonging to hydrolyzable tannins, different phenolic acids (e.g., protocatechuic, ellagic and gallic acids), and flavonols (e.g., quercetin glucuronide). The heterogeneity of the phenolic compounds present in the extract makes it valuable from a therapeutic point of view. The microLC-TOF-MS analysis confirmed the presence and enabled the quantitative determination of the most commonly reported constituents of *O. paradoxa* extracts with catechin and its gallate (11.2 and 1.9%), penta-*O*-galloyl-β-d-glucose (1.8%) as well as phenolic acids (gallic and ellagic acids) present in principal amount (37.3 and 24.7%, respectively). So far, several reports revealed beneficial effects of particular polyphenols, among which phenolic acids (especially gallic and ellagic acids), as well as quercetin and (epi)catechin, are of the most significant interest. Recent studies strongly pointed out the anti-metastatic properties of phenolic acids. GA decreased matrix metalloproteinase-2 and -9 both at gene and protein level, resulting in the inhibition of migration and invasion capacity of melanoma cells, prostate, colon cancer, and glioma cells [9,10,12,15]. Several studies investigated the activity of the *O. paradoxa* extract in respect to penta-*O*-galloyl-β-d-glucose (PGG) [2,46], another bioactive compound that was shown to reveal anti-proliferative effects in hepatocellular carcinoma cell line [47]. Moreover, the conjugation of PGG with cisplatin enhanced the antitumor effect of chemotherapy in renal cancer cells [48]. We postulate that the presence of PGG in the composition of our extract, even in the minor concentration, enriches the value of all biologically active compounds. Although we did not detect epigallocatechin gallate (EGCG) alone in our extract, other compounds belonging to the EGCG family, like, epicatechin gallate and catechin, were identified in our extract in dominant amount. Altogether, the composition of the EPE, rich in phenolic acids (gallic and ellagic acid), catechin and its derivative epicatechin gallate, as well as the presence of PGG, make the extract a valuable antitumor agent. Referring to the last decade’s data on the virtue of polyphenols in anticancer therapy, the application of their combined mixtures seems reasonable [49].

In the present work, both anti-proliferative effect and anti-invasive properties of the standardized extract obtained from defatted seeds of *Oenothera paradoxa* against malignant pleural mesothelioma cells were studied. MPM is recognized as a highly aggressive tumor of pleura, arising in response to prolonged asbestos fiber exposure. It engages multiple inflammatory processes in the mesothelial cells, which lead to tumor cell promotion, uncontrolled proliferation, increasing invasiveness ability, and finally metastasis. The latency period affects early recognition and treatment options. A majority of patients diagnosed at an advanced late stage cannot undergo pleura resection, and unfortunately, poorly respond to systemic chemotherapy [27]. Recent studies consider a single use of particular compounds in cancer treatment, including malignant pleural mesothelioma. The mechanisms of polyphenols action are still being thoroughly investigated, although for most of them, the observed cancer cell death is triggered by targeting molecular pathways directly involved in tumorigenesis and modulation of inflammatory processes. In the light of several encouraging reports on the beneficial effects of single polyphenols, including resveratrol, quercetin, curcumin, and epigallocatechin gallate [50,51,52,53] in malignant mesothelial cells treatment, we decided to test a mixture of polyphenols extracted from *O. paradoxa* seeds. The influence of polyphenol extracts from evening primrose on cancer proliferation has been studied before [29,30,41,54]. However, this is the first time that anticancer activity was studied in malignant pleural mesothelioma cells.

The extract not only strongly inhibits the viability of these cells but also reveals anti-metastatic activity. To our surprise, the extracts revealed a specificity against invasive cells. Our study disclosed that the MSTO-H211 and JU77 cells that showed higher invasiveness potential were four times more sensitive to EPE than the NCI-H28 cells with lower invasiveness potential. This phenomenon may have profound significance when searching for new therapies against the malignant pleural mesothelioma, an aggressive cancer with one of the worst prognoses in advanced stages. The five-year survival decreased from 20% in the first stage to 8% in the fourth stage of MPM development [55]. To confirm the observation of an increased EPE sensitivity of cells with high invasive ability, we checked whether cell proliferation inhibition had its consequences in cell cycle regulation. Indeed, we demonstrated that EPE caused a dose-dependent accumulation of JU77 cells in the G2/M phase, whereas EPE effect on NCI-H28 cells was almost undetectable.

It should be noted that essential components of polyphenols fraction within EPE contain ellagic acid, gallic acid, and catechin, which are involved in cell growth arrest by eliciting the G2/M phase of the cell cycle [8,16]. The blockade of individual phases of the cycle is also often accompanied by an increase in the number of cells undergoing apoptosis [16], which has also been confirmed in our research. We presume that the EPE-dependent inhibition of MPM cell invasiveness might be the effects of the unique polyphenol composition of EPE. It should be emphasized that EPE extract is characterized by a high concentration of ellagic acid and gallic acid [10], the well-described anti-metastatic components, that could support the EPE function.

It has been revealed that the extract of aerial parts of *O. biennis* inhibited the migration abilities of human melanoma cell lines A375 in a concentration-dependent manner [56]. In contrast, oral supplementation of oil from evening primrose (EPO) favors skin regeneration via increasing wound healing properties in dermal cells. However, this role is attributed to gamma-linolenic acid presented in EPO [57,58]. Subsequently, we decided to assess the impact of EPE on wound healing in MPMs cell lines. In this case, EPE treatment induced only slightly marked inhibition of collective cell migration in highly sensitive EPE cell lines (MSTO-H211 and JU77) and not-significant alteration in the non-sensitive EPE cell line. We suppose that the minor inhibition of migration observed in MPM cells could be the result of cell-cell interaction that modulated cohesive cell movement [59].

Previous studies demonstrated that particular fractions of evening primrose extract, e.g., flavanols, might regulate cancer invasiveness via downregulation of matrix metalloproteinases expression and secretion (MMPs) [41,54,59]. Our studies showed that the EPE inhibited MMP-7 secretion in EPE-sensitive MPM cells (JU77 and MSTO-H211). We observed that a non-toxic concentration of EPE (100 µg/mL) inhibited about three times as much MMP-7 secretion. These results are convergent with the results observed by several authors in colon, breast, and prostate cells [22,41,54,59,60]. Moreover, we observed that the process was associated with a robust (about 80%) inhibition of cell invasion. Although the invasion ability was demonstrated previously in prostate cells, the effect observed in that studies indicated only a 27% decrease of invasiveness. Surprisingly, it was lower than in normal cells [60]. We postulate that the observed process might be the effect of the unique composition and synergic activity of EPE or high level of gallic and ellagic acids, two polyphenols characterized by high anti-metastatic properties.

## 5. Conclusions

Our research demonstrates for the first time that the extract obtained from evening primrose (EPE) might be an essential factor in the prevention and treatment of MPM. We observed that the EPE extract shows not only anti-cancer properties manifested by inhibition of proliferation, blockade in the G2/M phase of the cell cycle, and induction of apoptosis but also has anti-metastatic properties. EPE caused the inhibition of cell migration and invasion, as well as the inhibition of MMP-7 secretion, which is associated with increased invasion capacity. The uniqueness of extract isolation, its composition, and its anti-tumor activity suggest that it might be used as an adjuvant in anti-MPM treatment and could increase the survival of patients with an invasive form of MPM. Notably, the stability of EPE extract and the availability of the raw material for its isolation allows for the fast and non-complicated implementation of EPE in the pharmaceutical industry.

## Figures and Tables

**Table 1:** Characterization of the polyphenol extract from defatted evening primrose seeds.

Spectrophotometric Analysis (n = 3) ^A^						
Total Polyphenols Content (mg/g)			208.24 ± 8.92			
Qualitative microLC-TOF 4600 MS Analysis ^B^						
No. ^a^	t_R_ (min) ^b^	Assigned identity ^c^	MW ^d^	[M − H]¯ (*m*/*z*) ^e^	Chemical Entity ^f^	MS/MS Ions (*m*/*z*) ^g^
1.	0.52	**Gallic acid**	170.12	169.0134	Phenolic acid	125.0235, 107.0136
2.	0.52	**Caffeic acid**	180.16	179.0546	Phenolic acid	161.0432, 119.0325, 89.0238
3.	0.53	Digalloyl glucose	484.37	483.0782	Hydrolyzable tannins	331.0660, 313.0562, 169.0136
4.	0.58	**Procyanidin dimer**	578.52	577.1369	Flavan-3-ol	451.1028, 425.0867, 289.0706
5.	0.91	**Catechin**	290.26	289.0708	Flavan-3-ol	245.0805, 109.0292
6.	0.98	Quercetin pentoside	434.09	433.1120	Flavonols	343.0803, 181.0496
7.	1.06	Protocatechuic acid	154.12	152.9175	Phenolic acid	135.9133, 122.0367, 109.0291
8	1.10	Procyanidin tetramer	1154.36	1153.2692	Flavon-3-ol	863.1865, 575.1145, 287.0532
9	1.16	Procyanidin trimer	866.21	865.1989	Flavan-3-ol	695.1420, 577.1371, 287.0514
10	1.20	Procyanidin trimer gallate	1018.34	1017.2184	Flavan-3-ol	865.2041, 287.0569
11.	1.21	**Quercetin/Ellagic acid**	302.24/302.20	300.9980	Phenolic acid	286.0818, 257.0354, 185.0230
12.	1.28	**Penta-*O*-galloyl β-d-glucose**	940.68	939.1179	Hydrolyzable tannins	787.1009, 769.0939, 617.0820
13.	1.32	Procyanidin dimer gallate	730.15	729.1476	Flavan-3-ol	577.1382, 559.1232, 407.0792, 289.0707
14.	1.64	Tetragalloyl glucose	788.60	787.1025	Hydrolyzable tannins	617.0801, 465.0645, 295.0421
15.	1.71	Methyl gallate	184.15	183.1025	Phenolic acid	139.1131, 111.0821

(**A**) Total polyphenols content in *O. paradoxa* extract determined by Folin–Ciocalteu method as gallic acid equivalents, expressed in mg per g of dry extracts; the value represents means of three independent experiments ± standard deviation. (**B**) Qualitative identification of particular compounds in EPE by microLC-TOF 4600 MS analysis. a: The ordered number assigned to compounds’ occurrence during microLC-TOF-MS analysis and retention times; b: retention time; c: assigned identity based on currently reported constituents of evening primrose extracts; d: approx. molecular weight (the values are rounded to the nearest integer); e: parent molecular ion in negative ionization mode; f: chemical entity to a particular group of polyphenols; g: tentative identities of the compounds based on peak fragmentation ions (MS/MS spectra) detected in *Oenothera paradoxa* extract (EPE24). MS/MS spectra present in Figure S2 in Supplementary Information. Bolded compounds were further quantitatively determined by LC-TripleTOF 5600^+^-MS analysis.

**Table 2:** Quantitative identification of particular compounds in defatted evening primrose seeds extract determined by LC-TripleTOF 5600^+^-MS based on chromatograms present in Figure S3 in Supplementary Information; RT-retention time, *m*/*z*-mass to ion ratio of compounds in negative (#) and positive (*) ionization mode of TOF 5600^+^-MS, mg/g-quantitative analysis of phenolic compounds according to standard curves.

		LC-TripleTOF-MS Analysis (n = 3)			
Compound	Structure		RT	*m*/*z*	mg/g
gallic acid	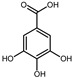		0.97	169.0501 ^#^	51.40 ± 0.13
catechin	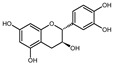		2.03	291.0917 *	23.23 ± 0.09
procyanidin B2	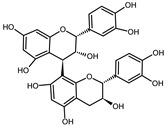		2.15	579.1384 *	0.98 ± 0.28
penta-*O*-galloyl-β-d-glucose	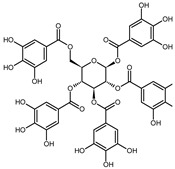		2.23	939.1206 ^#^	3.70 ± 0.06
ellagic acid	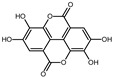		2.25	303.0145 *	77.67 ± 0.48
epicatechin gallate	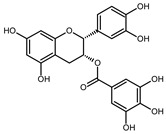		2.41	443.0941 *	3.96 ± 0.01
caffeic acid	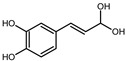		2.49	181.0501 *	1.31 ± 0.02

## Supplementary Materials

The following are available online at https://www.mdpi.com/2218-273X/10/11/1574/s1, Figure S1: Qualitative LC TripleTOF MS 4600 profile of EPE 24 performed in negative ionization mode, Figure S2: TripleTOF 4600 MS/MS product ions of particular compounds determined in EPE24 performed in negative ionization mode. Figure S3: LC-TripleTOF-MS 5600^+^ spectra of the selected compounds of *O*. *paradoxa* extract.
