# Supplementary material for: Polyphenol Extract from Evening Primrose (Oenothera paradoxa) Inhibits Invasion Properties of Human Malignant Pleural Mesothelioma Cells
Source: Biomolecules. 2020 Nov 19;10(11):1574. doi: 10.3390/biom10111574 (PMC7699585; doi:10.3390/biom10111574)
Supplement: Supplementary file 1 [file biomolecules-10-01574-s001.zip › Supplementary 1S_2S final.pdf]

Figure 1S. Qualitative LC-TOF-MS 4600 profile of EPE24 performed in negative ionization mode. 1 - gallic acid, 2 – catechin, 3 – quercetin/ellagic acid, 4 – procyanidin dimer gallate, 5 - tetragalloyl glucose. 6 – penta-O-galloyl-β-D-glucose, 7 – procyanidin trimer gallate, 8 – procyanidin tetramer

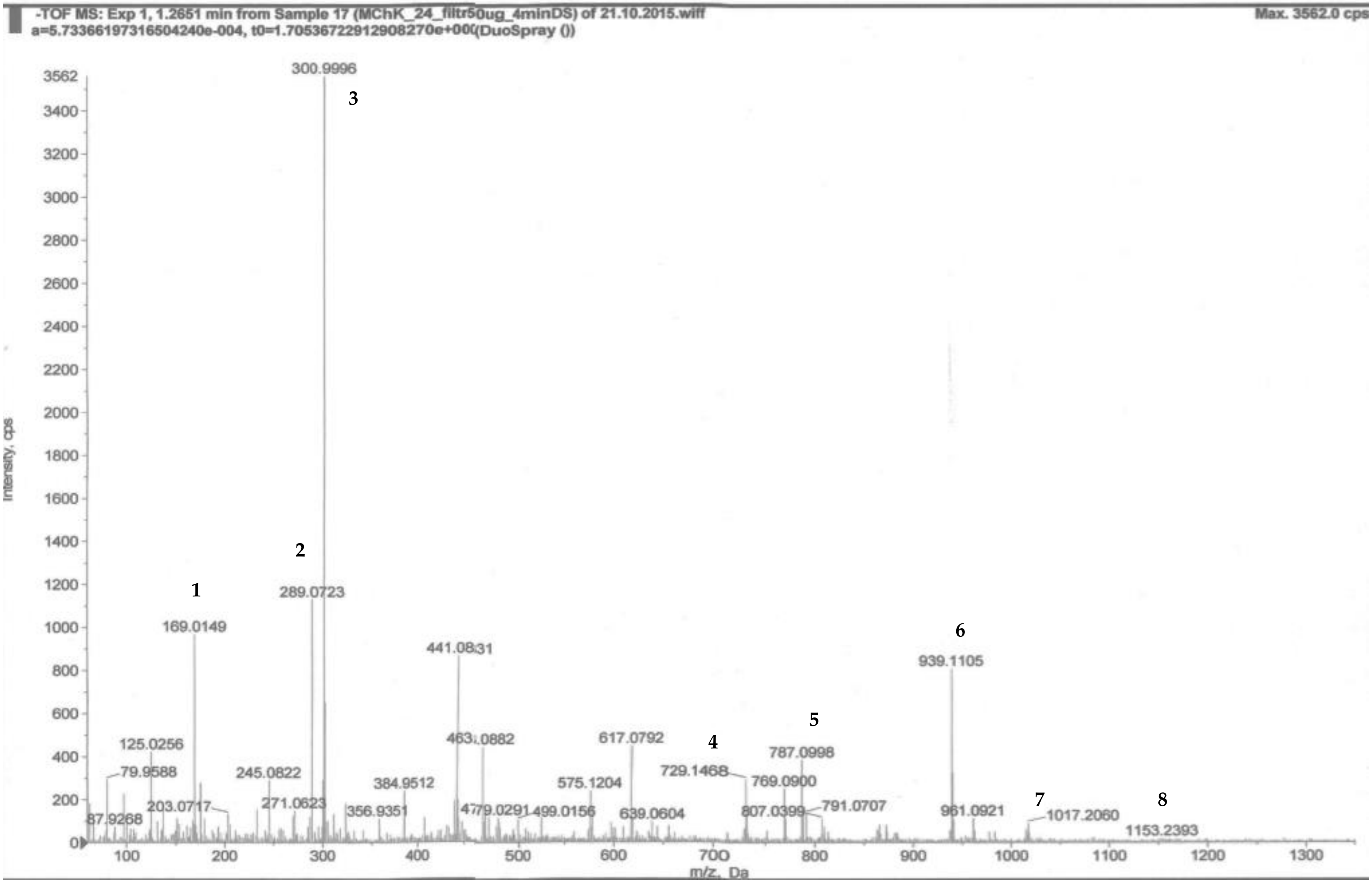

Figure 2S. TripleTOF 4600+ MS/MS product ions of particular compounds determined in EPE24 performed in negative ionization mode.

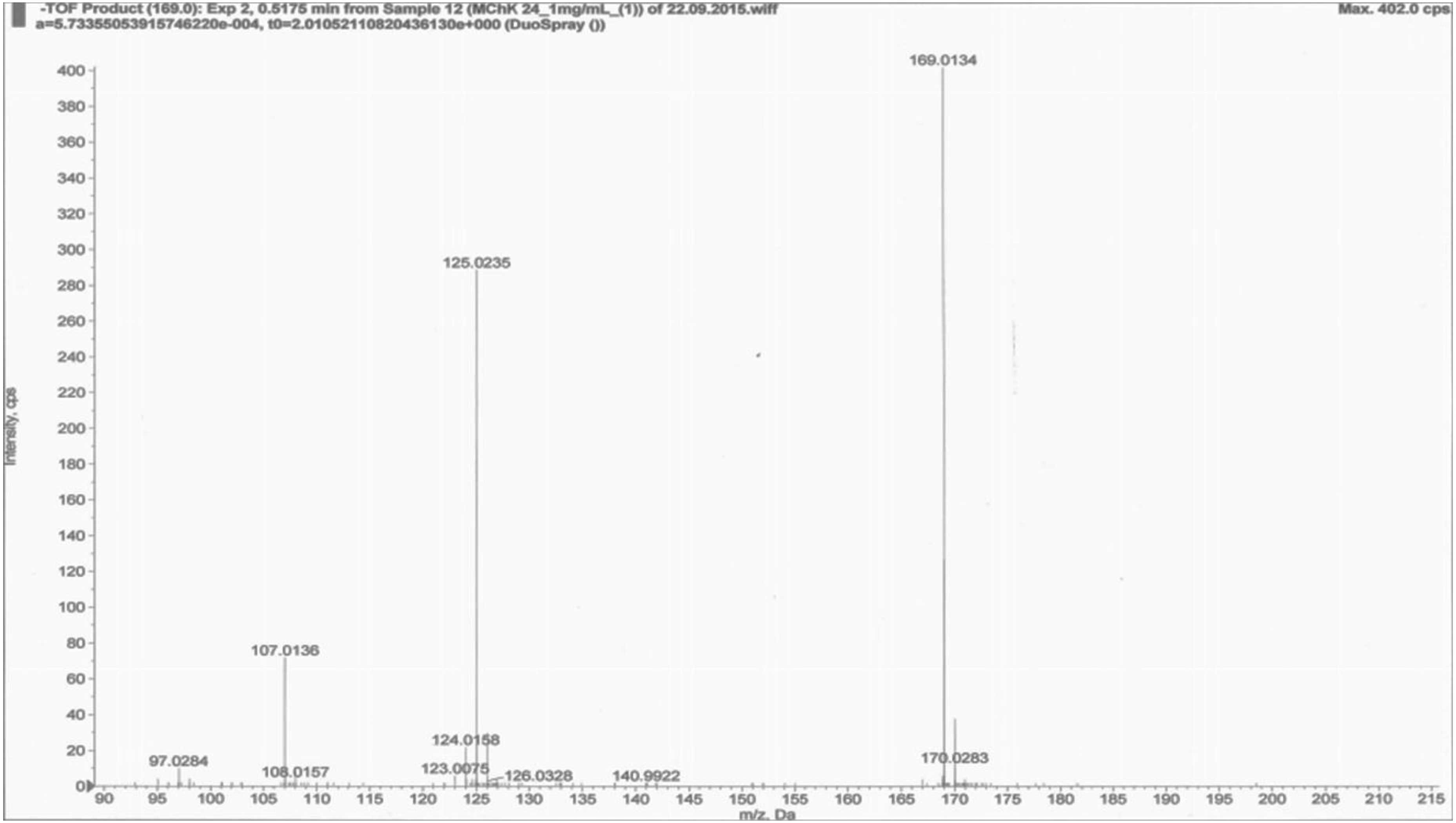

Gallic acid

Figure 2S. TripleTOF 4600+ MS/MS product ions of particular compounds determined in EPE24 performed in negative ionization mode.

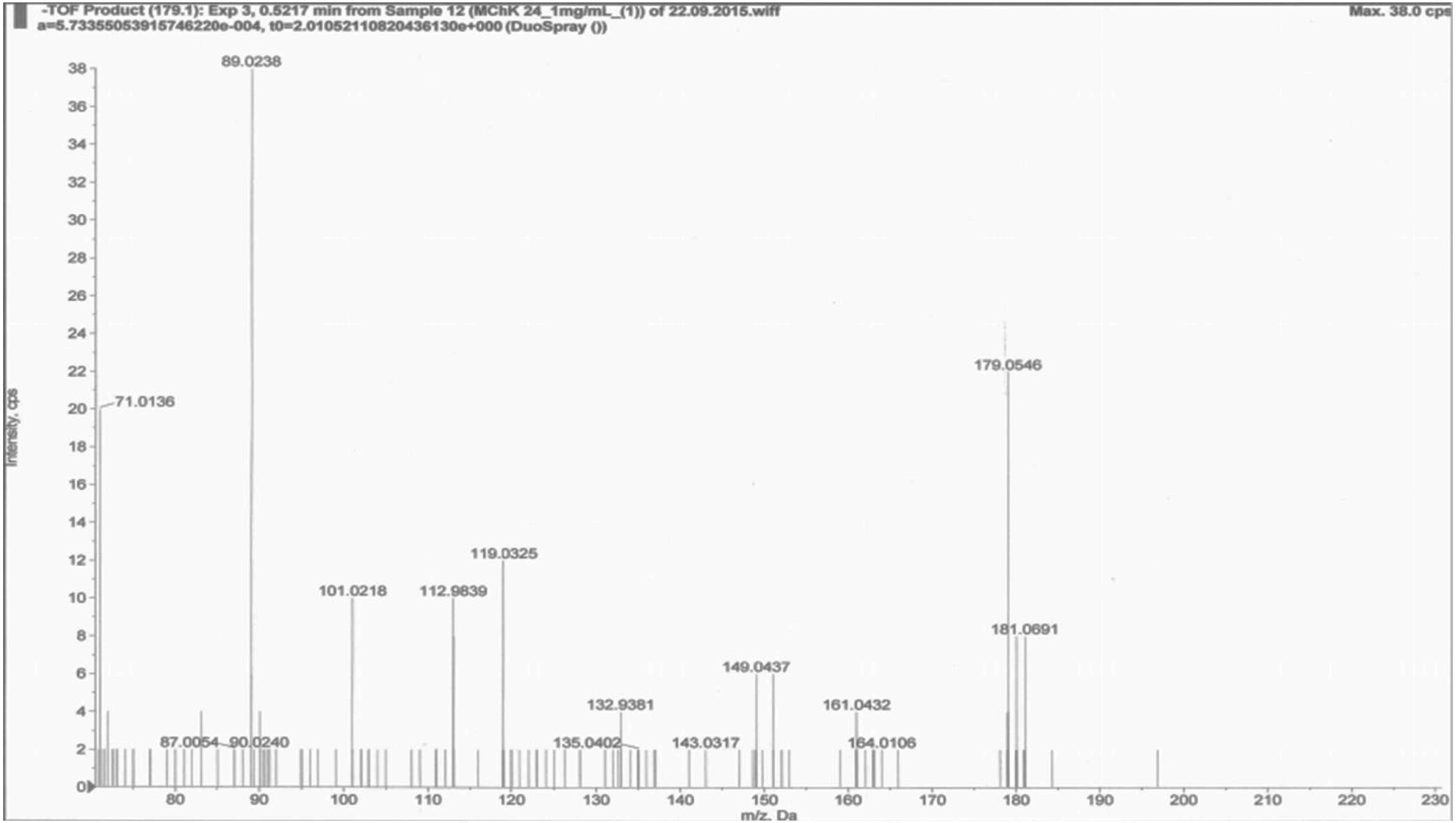

Caffeic acid

Figure 2S. TripleTOF 4600+ MS/MS product ions of particular compounds determined in EPE24 performed in negative ionization mode.

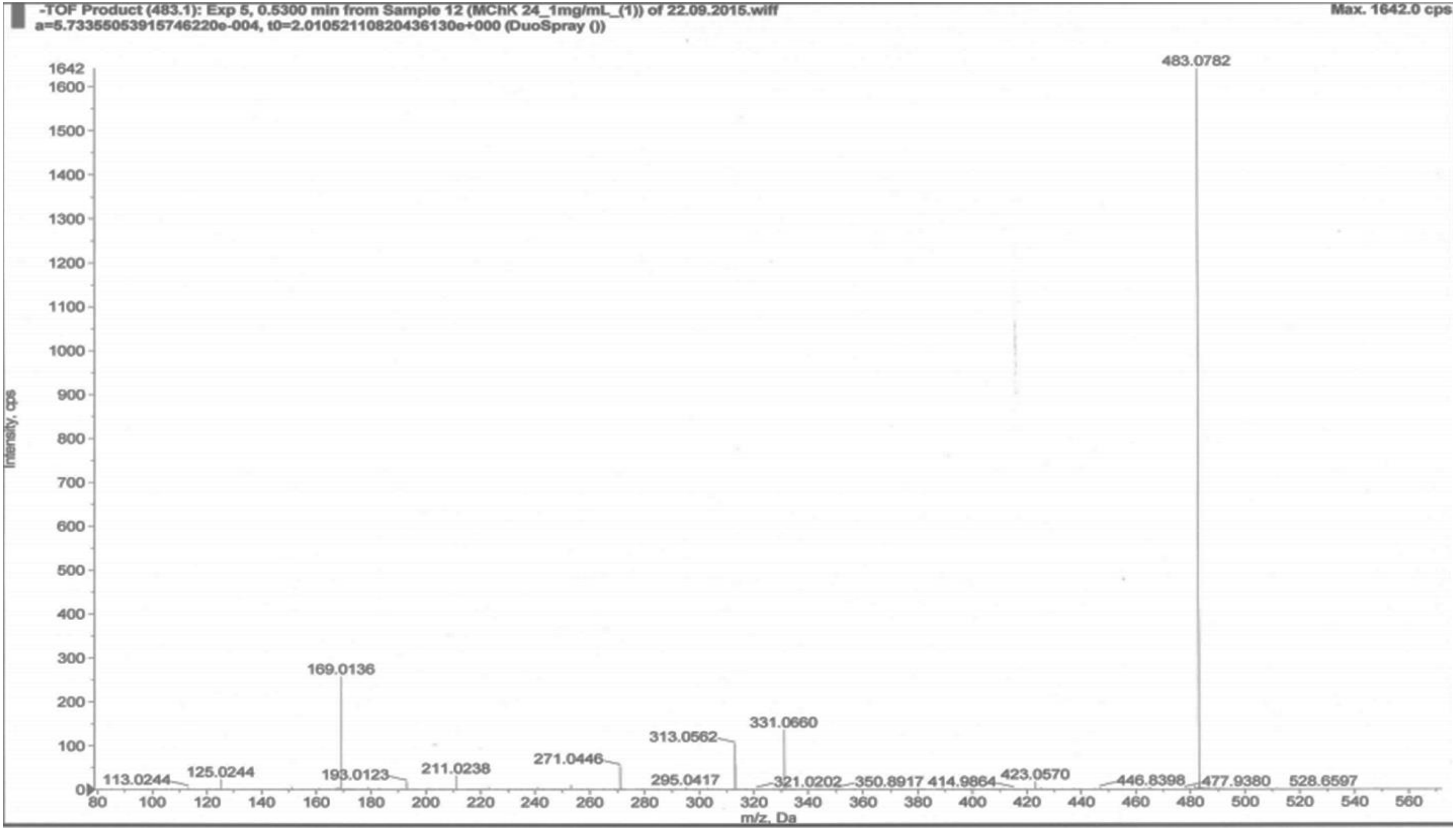

Digalloyl glucose

Figure 2S. TripleTOF 4600⁺ MS/MS product ions of particular compounds determined in EPE24 performed in negative ionization mode.

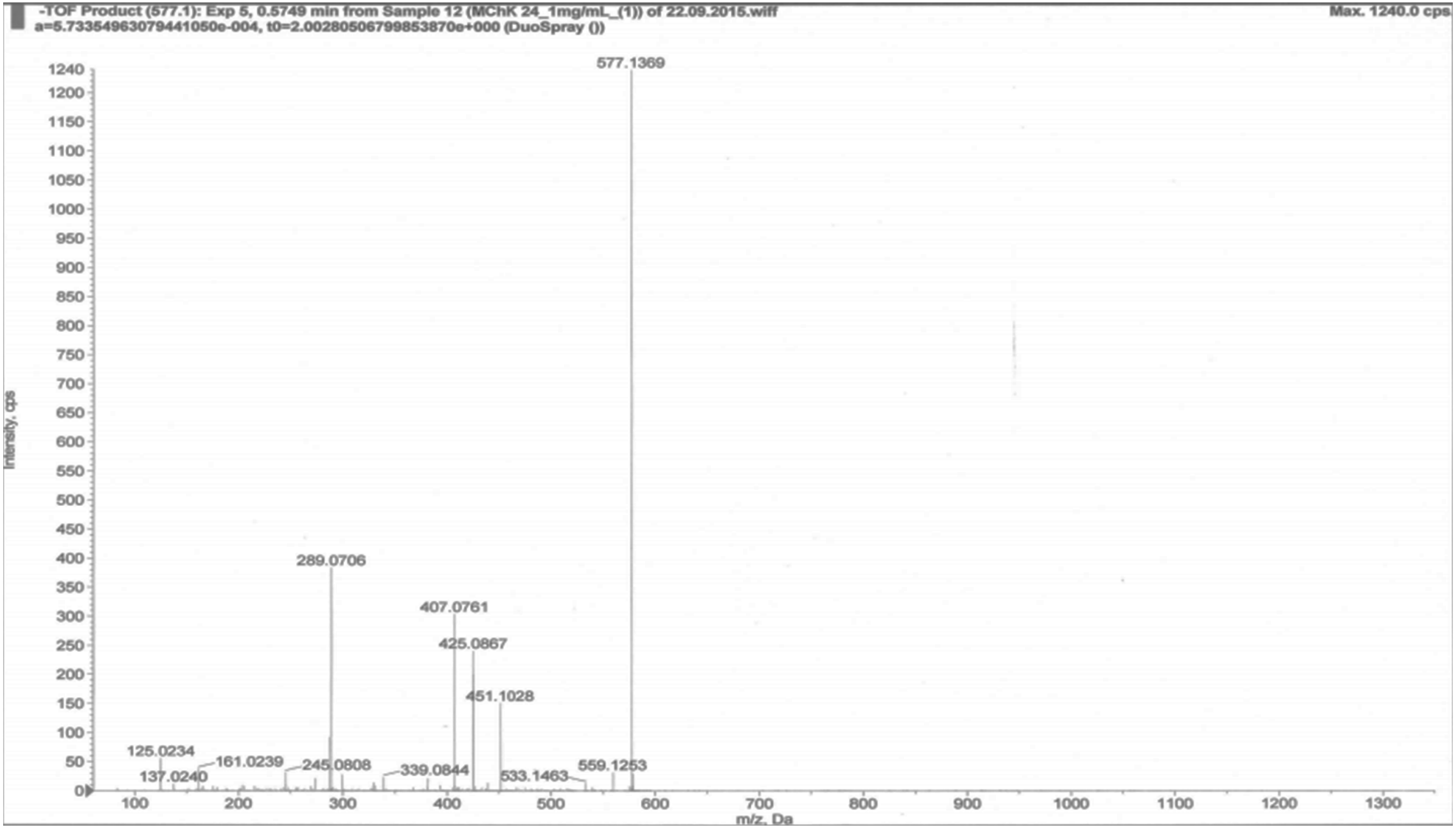

Procyanidin dimer

Figure 2S. TripleTOF 4600+ MS/MS product ions of particular compounds determined in EPE24 performed in negative ionization mode.

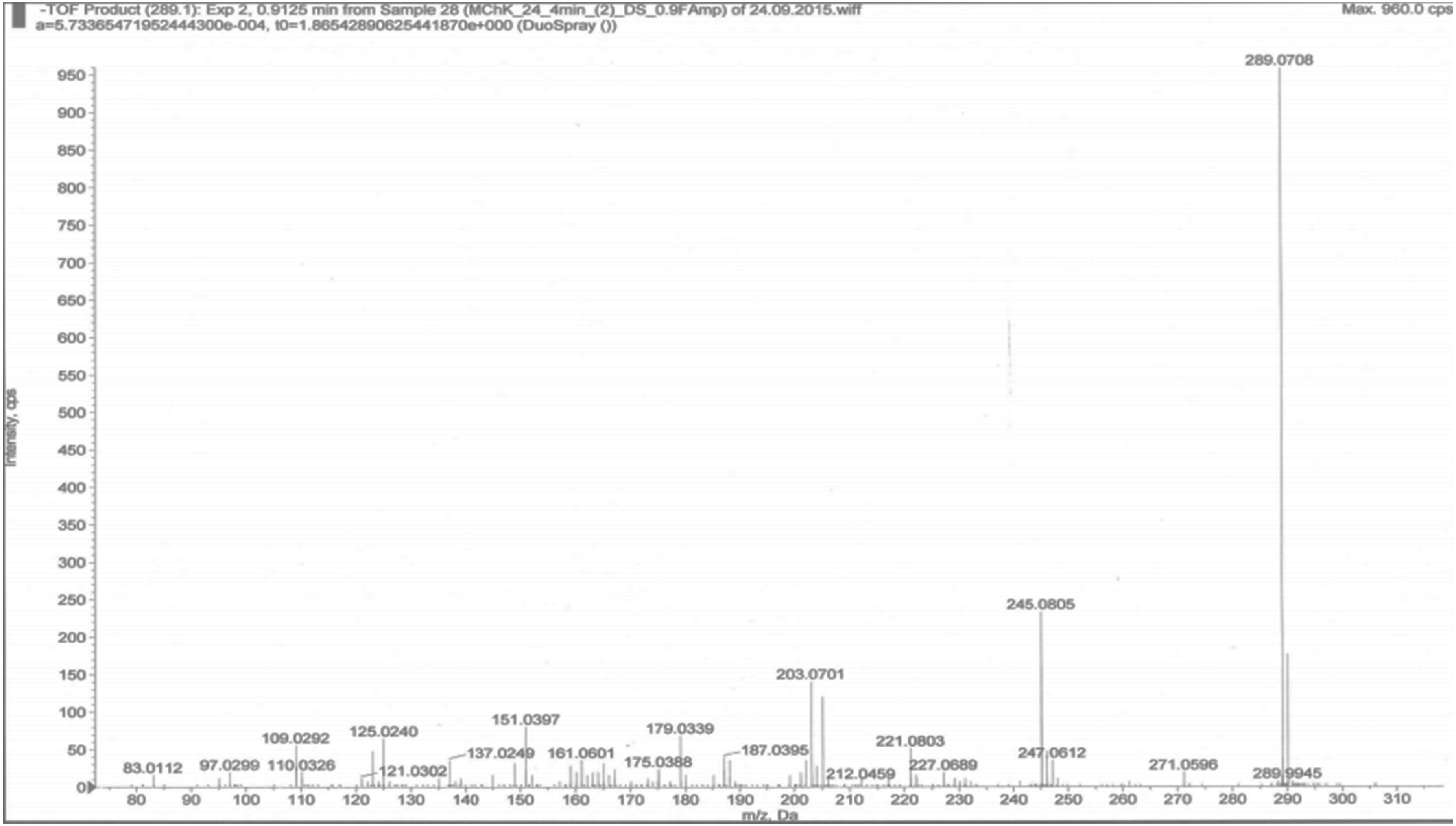

Catechin

Figure 2S. TripleTOF 4600+ MS/MS product ions of particular compounds determined in EPE24 performed in negative ionization mode.

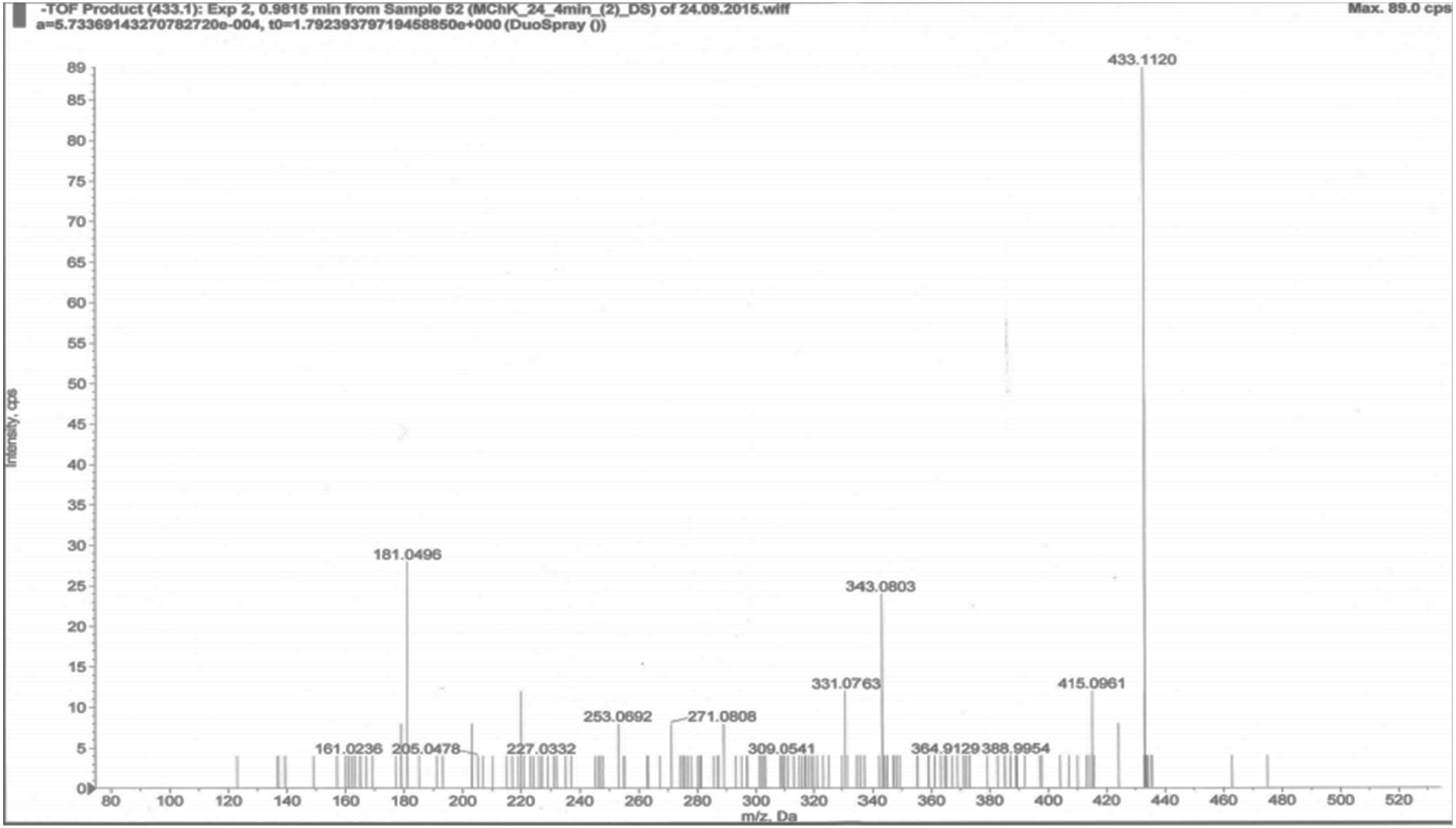

Quercetin pentoside

Figure 2S. TripleTOF 4600⁺ MS/MS product ions of particular compounds determined in EPE24 performed in negative ionization mode.

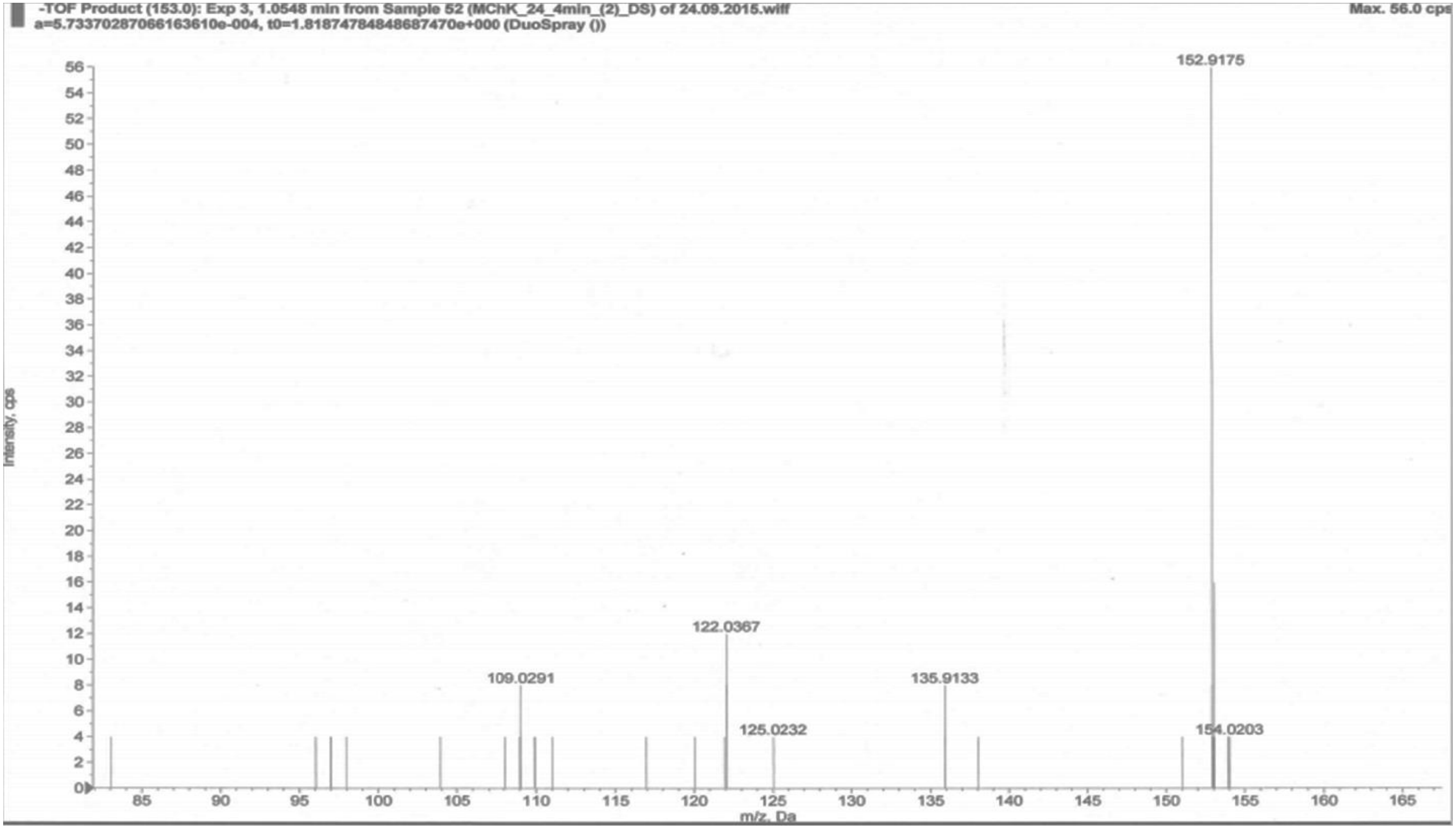

Protocatechuic acid

Figure 2S. TripleTOF 4600+ MS/MS product ions of particular compounds determined in EPE24 performed in negative ionization mode.

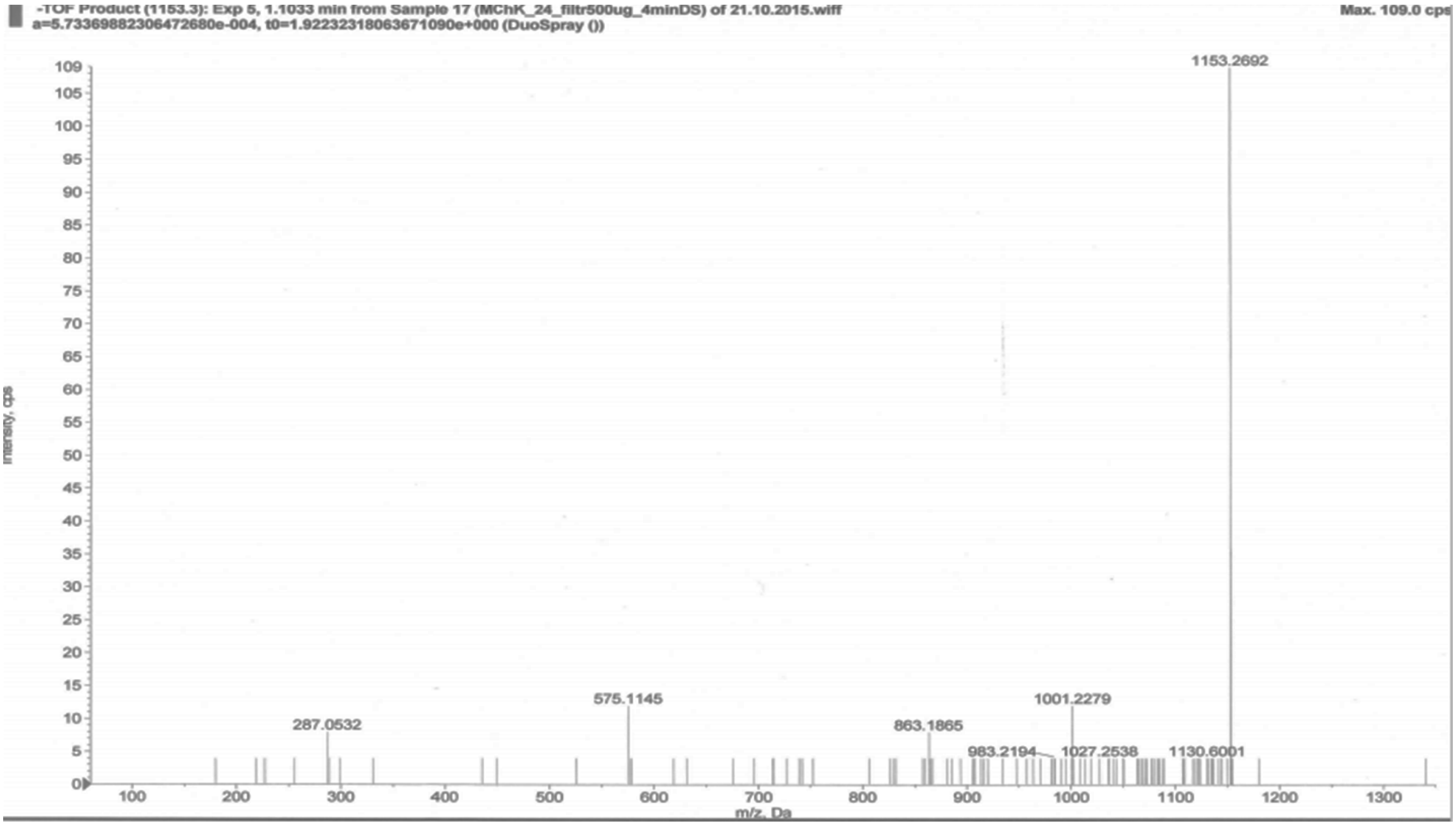

Procyanidin tetramer

Figure 2S. TripleTOF 4600+ MS/MS product ions of particular compounds determined in EPE24 performed in negative ionization mode.

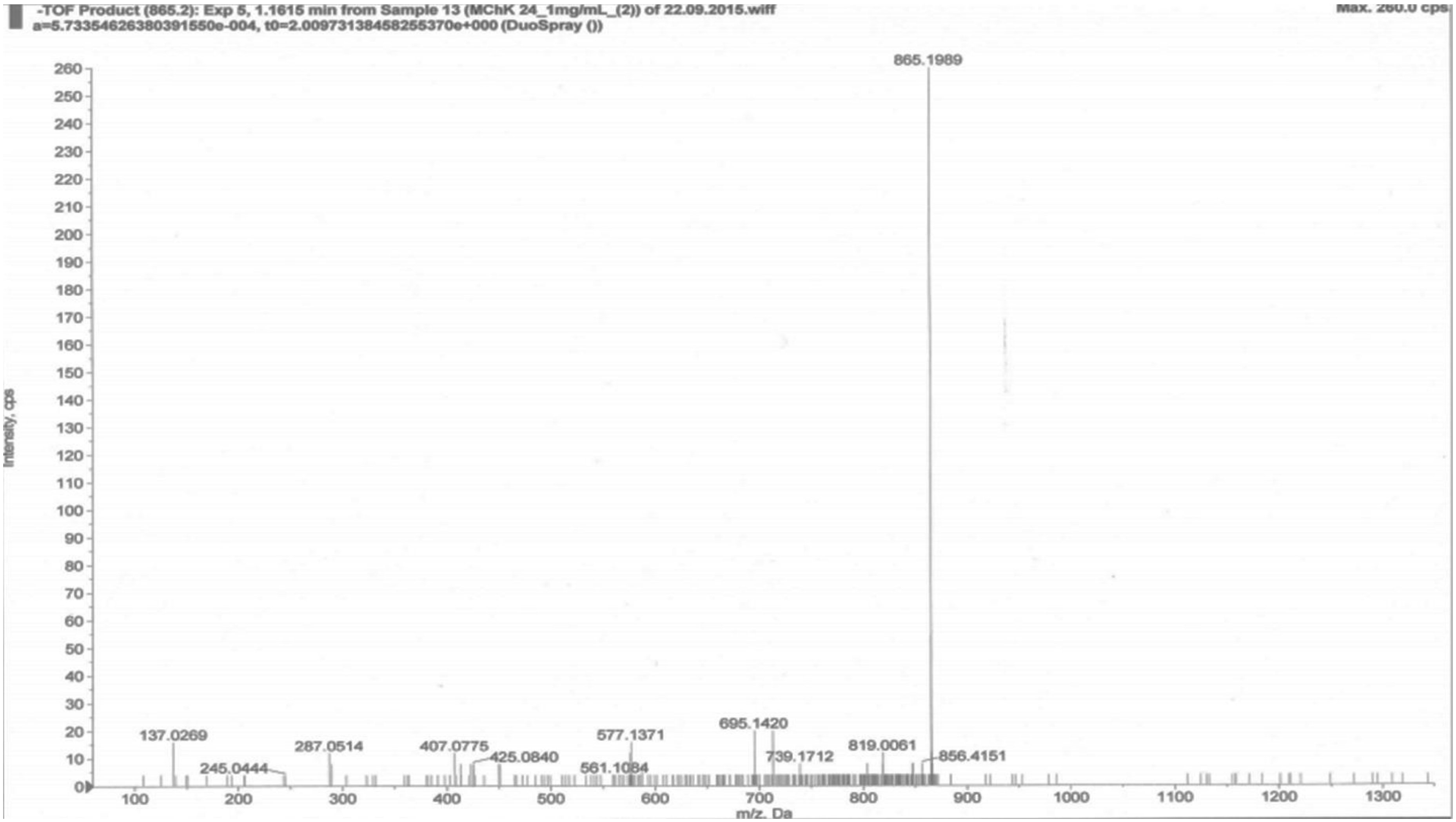

Procyanidin trimer

Figure 2S. TripleTOF 4600⁺ MS/MS product ions of particular compounds determined in EPE24 performed in negative ionization mode.

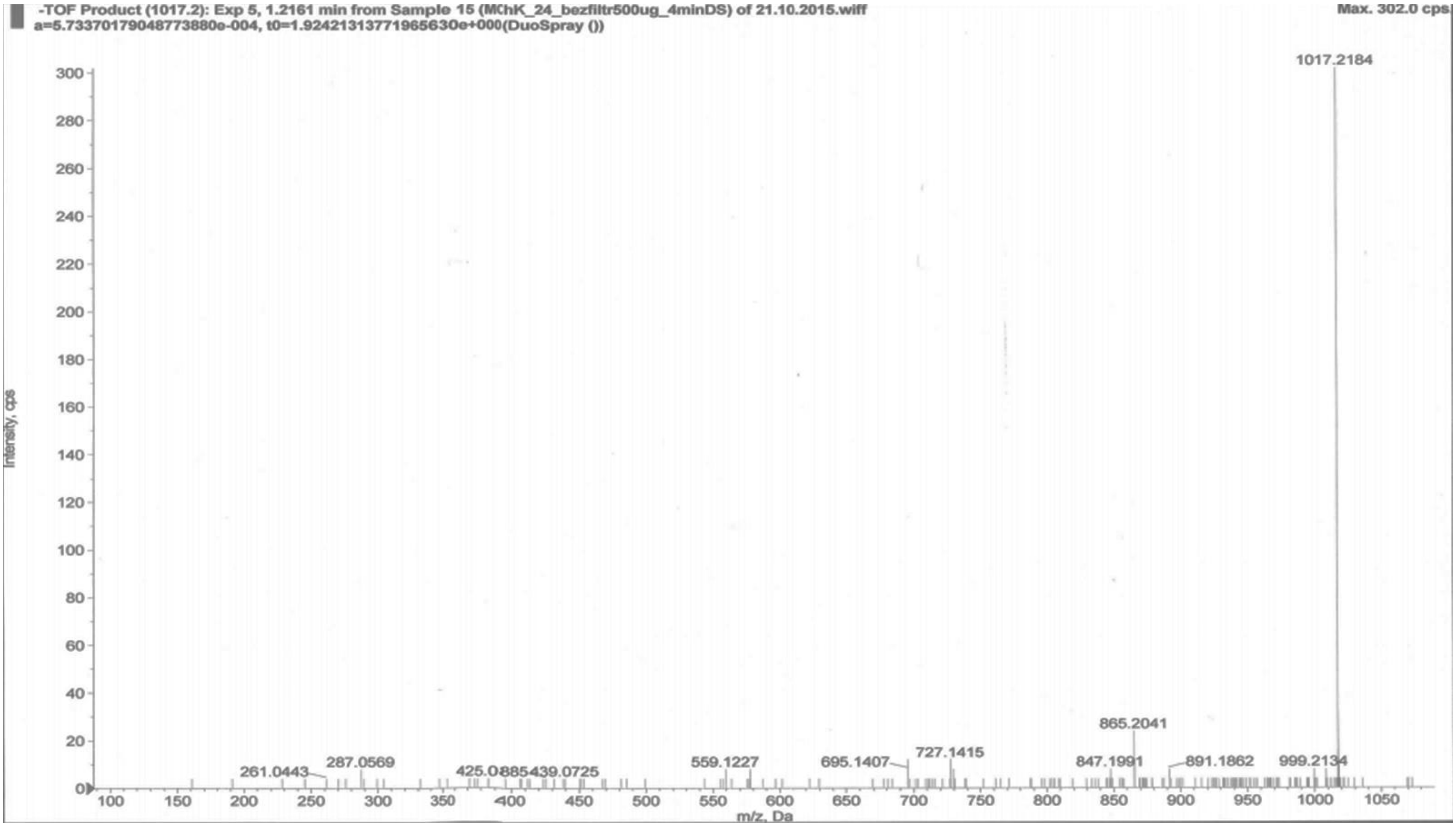

Procyanidin trimer gallate

Figure 2S. TripleTOF 4600+ MS/MS product ions of particular compounds determined in EPE24 performed in negative ionization mode.

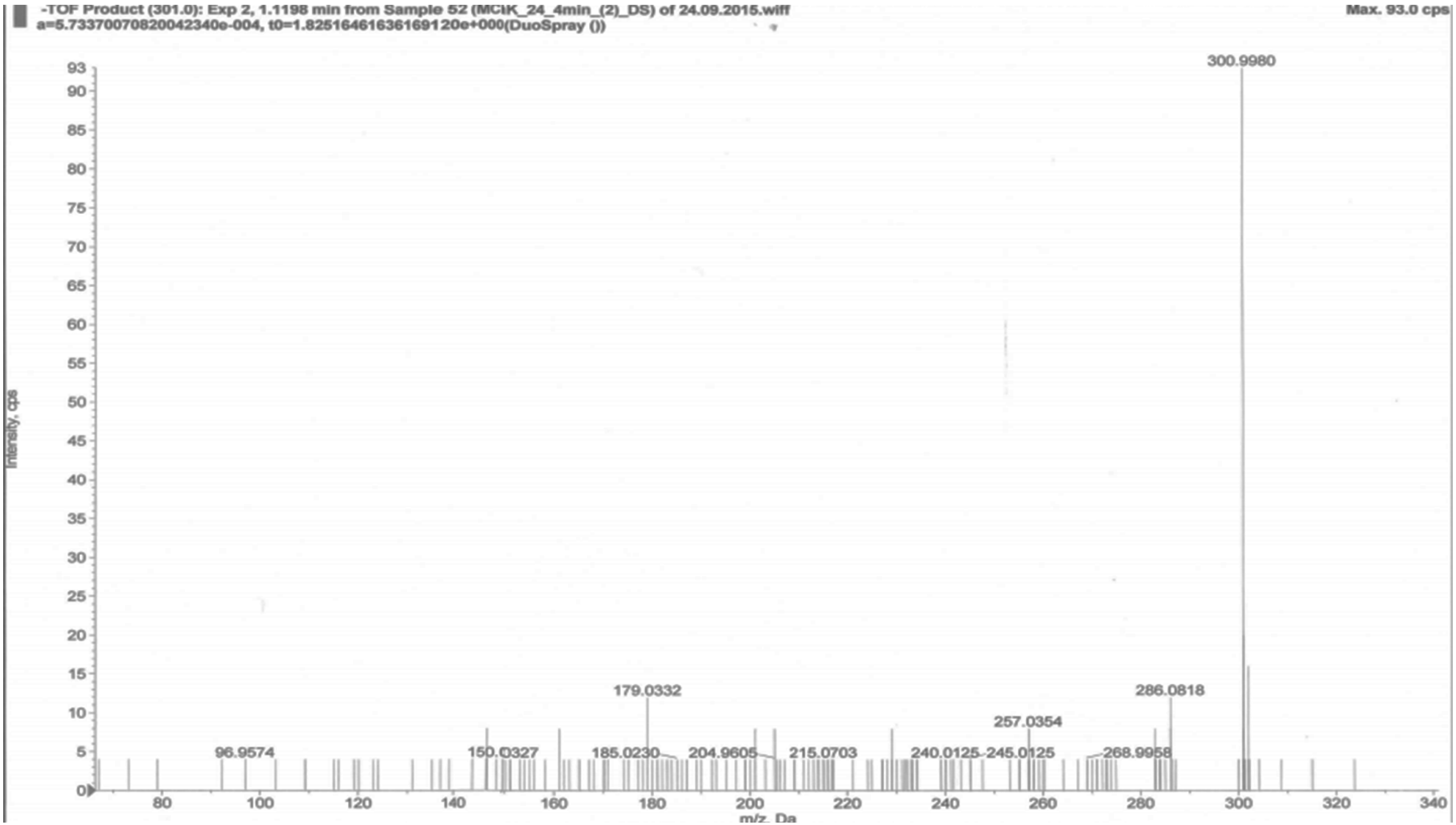

Ellagic acid

Figure 2S. TripleTOF 4600+ MS/MS product ions of particular compounds determined in EPE24 performed in negative ionization mode.

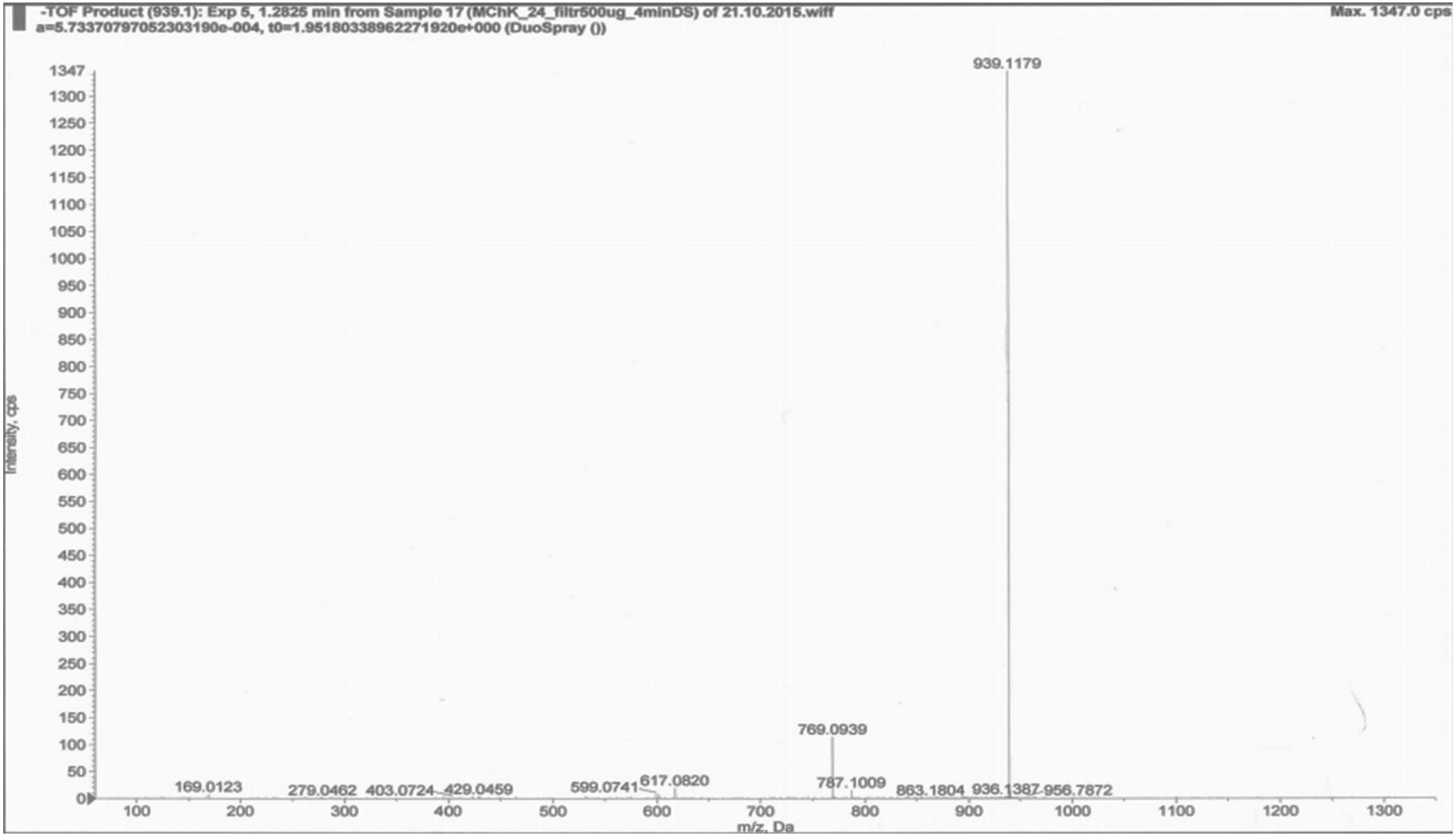

Penta-O-galloyl-β-D-glucose

Figure 2S. TripleTOF 4600+ MS/MS product ions of particular compounds determined in EPE24 performed in negative ionization mode.

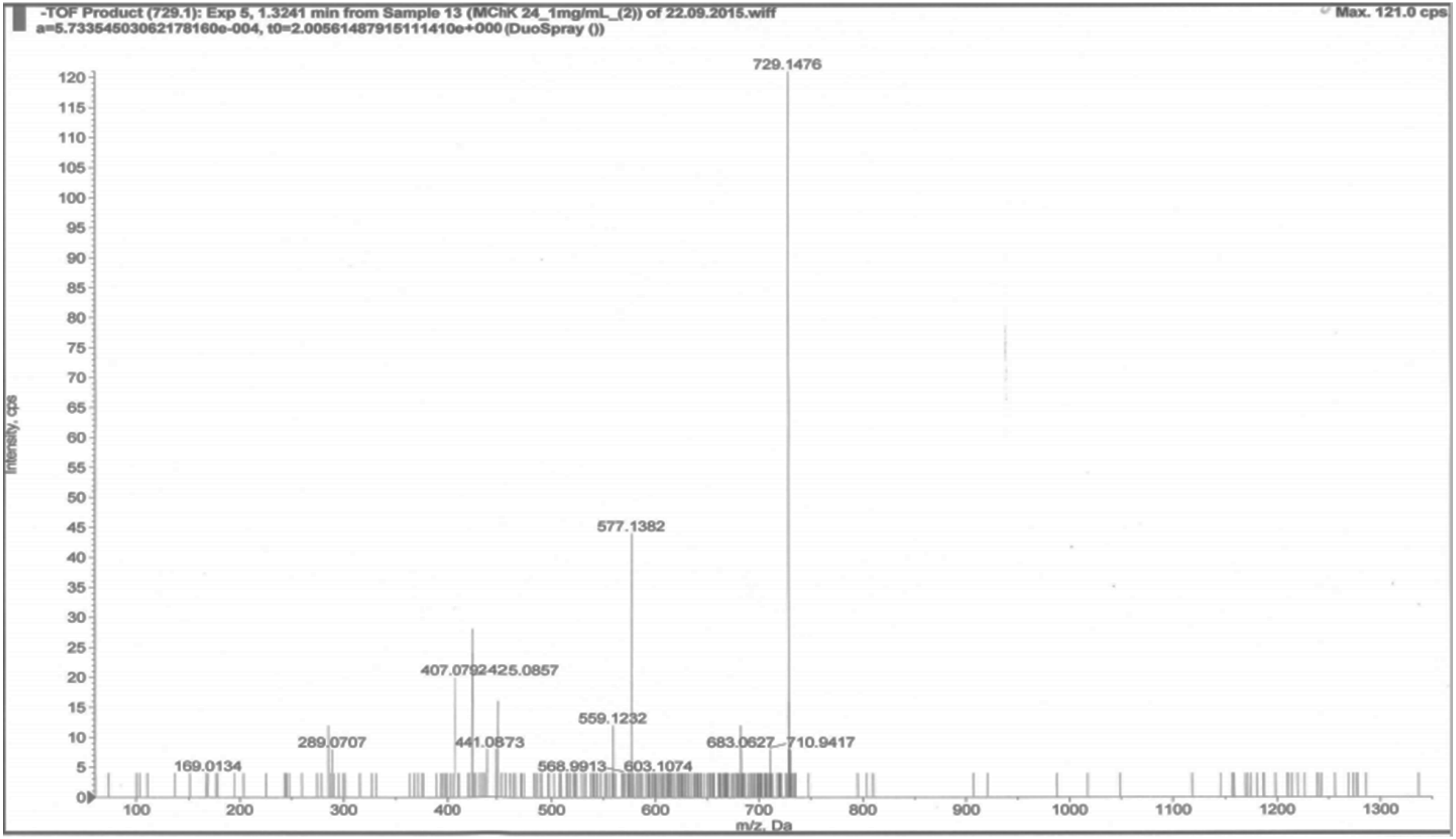

Procyanidin dimer gallate

Figure 2S. TripleTOF 4600+ MS/MS product ions of particular compounds determined in EPE24 performed in negative ionization mode.

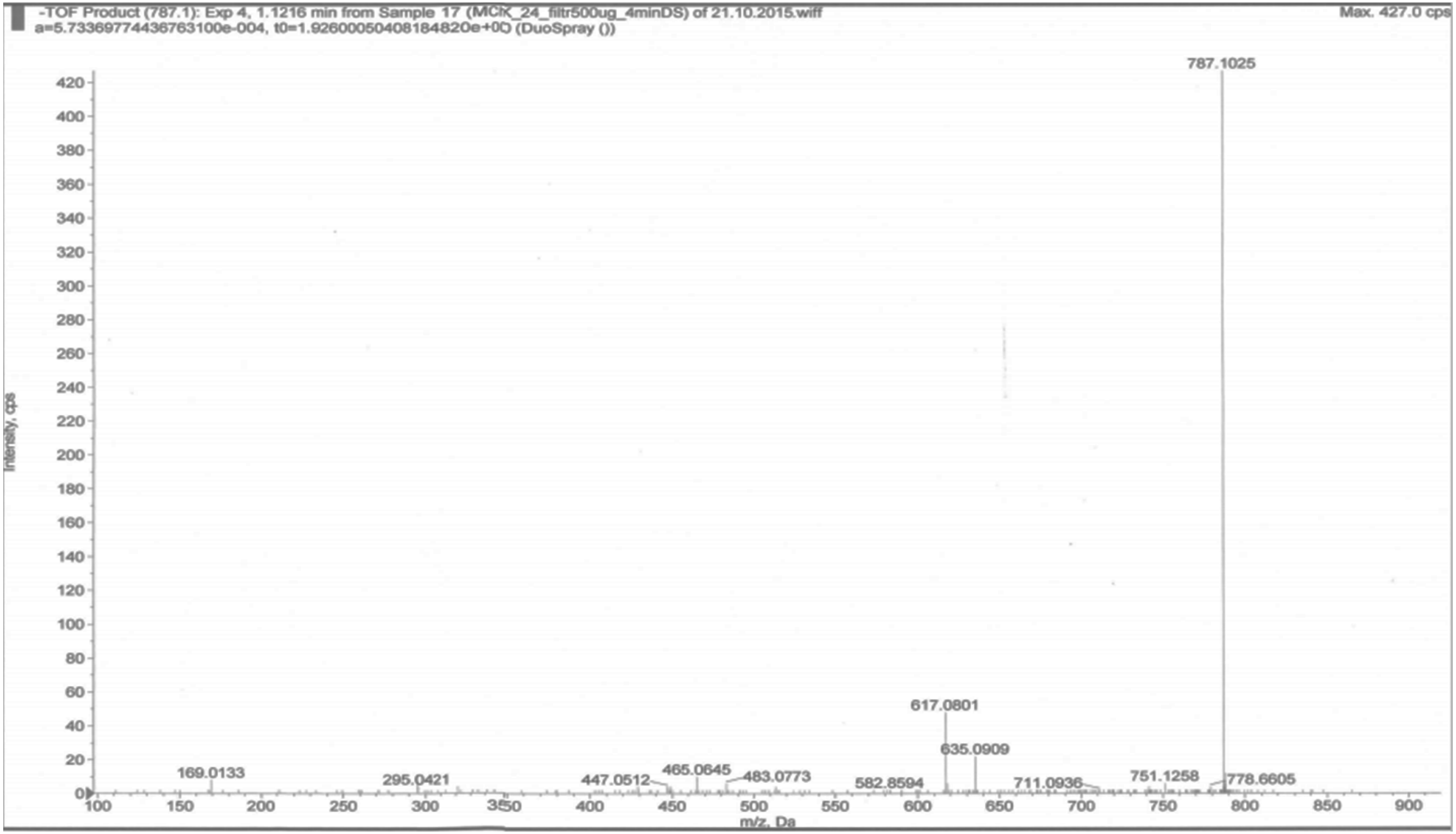

Tetragalloyl glucose

Figure 2S. TripleTOF 4600+ MS/MS product ions of particular compounds determined in EPE24 performed in negative ionization mode.

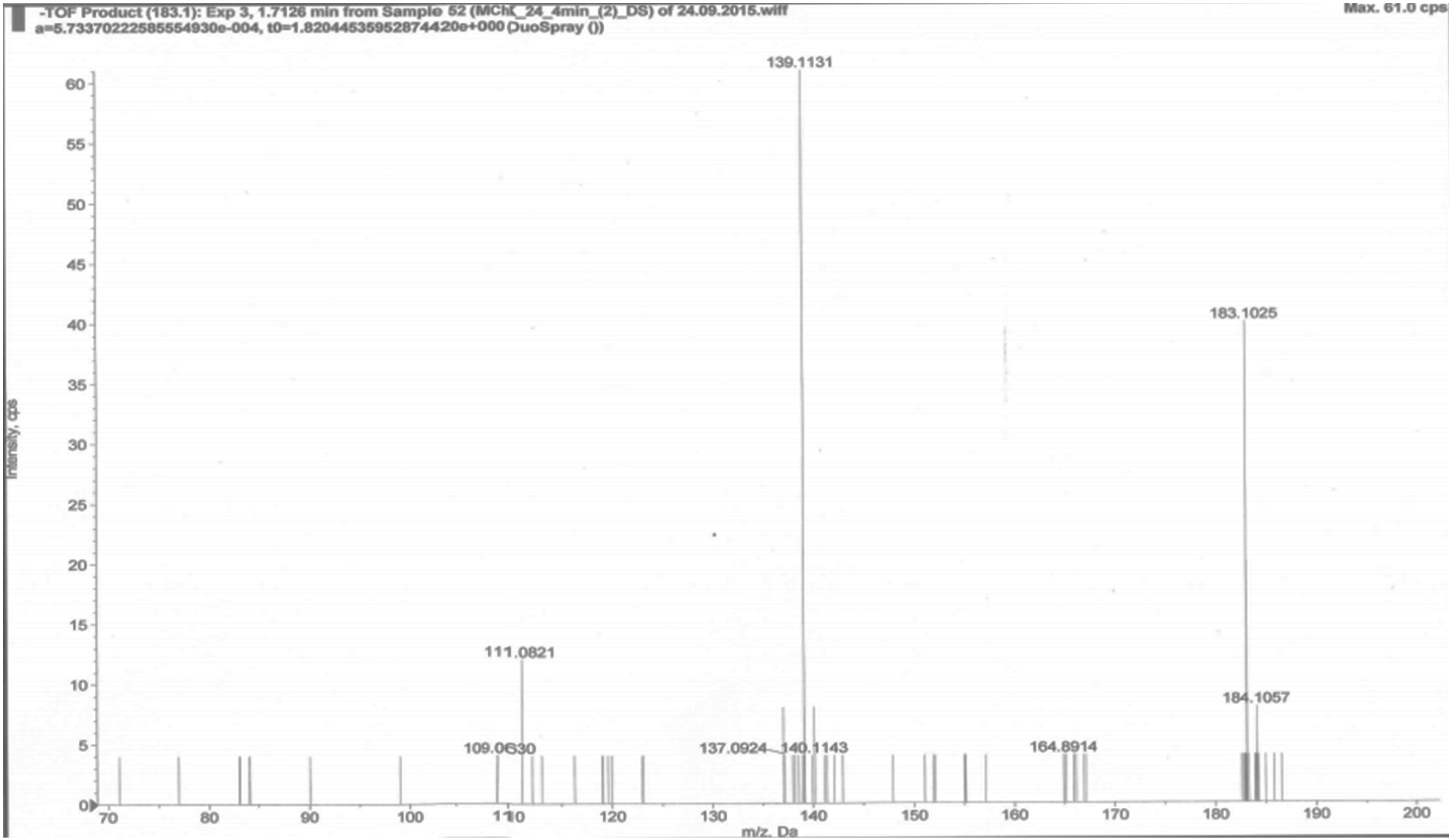

Methyl gallate
