# Supplementary material for: Polyphenol Extract from Evening Primrose (Oenothera paradoxa) Inhibits Invasion Properties of Human Malignant Pleural Mesothelioma Cells
Source: Biomolecules. 2020 Nov 19;10(11):1574. doi: 10.3390/biom10111574 (PMC7699585; doi:10.3390/biom10111574)

Figure 3S. LC-TOF-MS 5600+ spectra of the selected compounds of *O. paradoxa* extract. Phenolic profile of EP extract performed by LC-TripleTOF-MS in positive (A) and negative (B) ionization modes. 1. caffeic acid, 2. catechin, 3. epicatechin gallate, 4. quercetin , 5. ellagic acid, 6. procyanidin B2, 7. gallic acid, 8. PGG

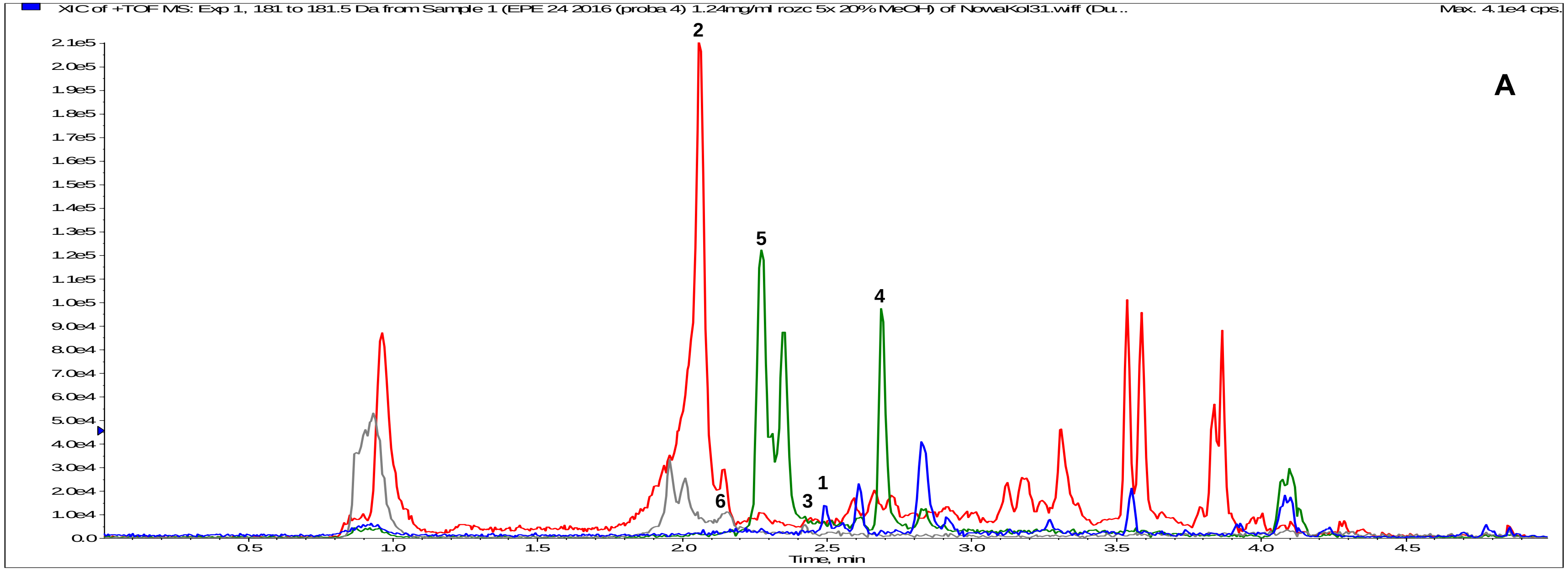

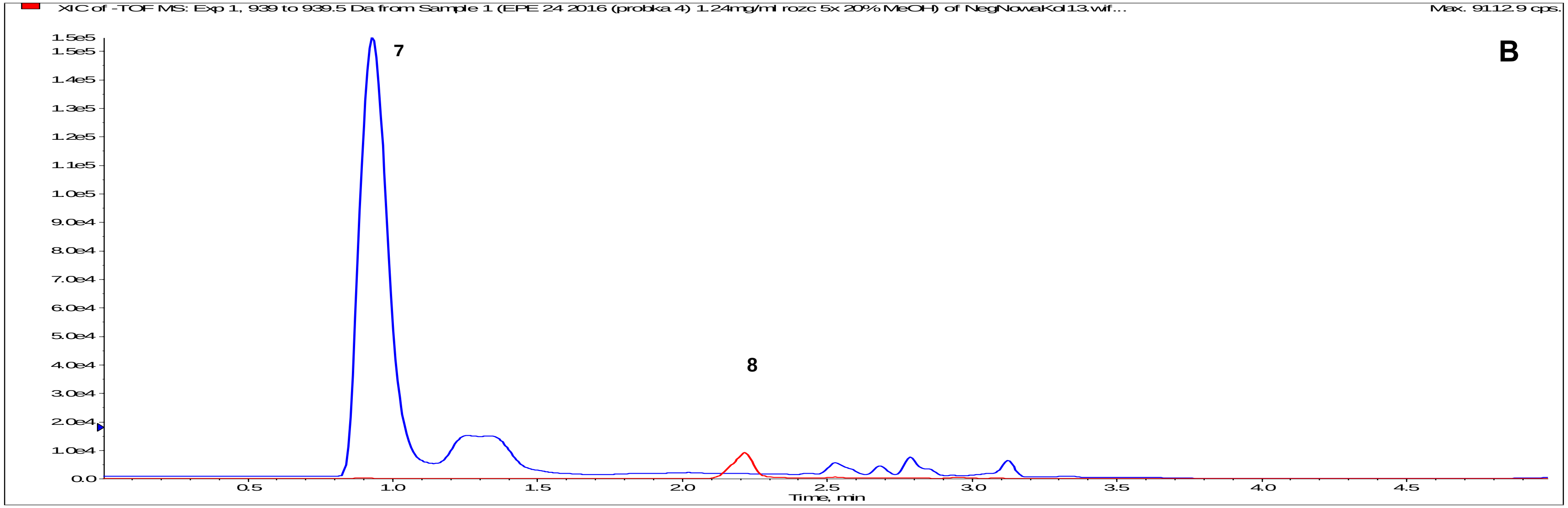

Supplement: Supplementary file 1 [file biomolecules-10-01574-s001.zip › Supplementary 3S final.pdf]
